# Small RNAs Originated from Pseudogenes: *cis*- or *trans*-Acting?

**DOI:** 10.1371/journal.pcbi.1000449

**Published:** 2009-07-31

**Authors:** Xingyi Guo, Zhaolei Zhang, Mark B. Gerstein, Deyou Zheng

**Affiliations:** 1 Institute for Brain Disorders and Neural Regeneration, Department of Neurology, Albert Einstein College of Medicine, New York, New York, United States of America; 2 Terrence Donnelly Centre for Cellular and Biomolecular Research, University of Toronto, Toronto, Ontario, Canada; 3 Department of Molecular Biophysics and Biochemistry, Yale University, New Haven, Connecticut, United States of America; 4 Department of Genetics and Department of Neuroscience, Albert Einstein College of Medicine, New York, New York, United States of America; Washington University in Saint Louis, United States of America

## Abstract

Pseudogenes are significant components of eukaryotic genomes, and some have acquired novel regulatory roles. To date, no study has characterized rice pseudogenes systematically or addressed their impact on the structure and function of the rice genome. In this genome-wide study, we have identified 11,956 non-transposon-related rice pseudogenes, most of which are from gene duplications. About 12% of the rice protein-coding genes, half of which are in singleton families, have a pseudogene paralog. Interestingly, we found that 145 of these pseudogenes potentially gave rise to antisense small RNAs after examining ∼1.5 million small RNAs from developing rice grains. The majority (>50%) of these antisense RNAs are 24-nucleotides long, a feature often seen in plant repeat-associated small interfering RNAs (siRNAs) produced by RNA-dependent RNA polymerase (RDR2) and Dicer-like protein 3 (DCL3), suggesting that some pseudogene-derived siRNAs may be implicated in repressing pseudogene transcription (i.e., *cis*-acting). Multiple lines of evidence, however, indicate that small RNAs from rice pseudogenes might also function as natural antisense siRNAs either by interacting with the complementary sense RNAs from functional parental genes (38 cases) or by forming double-strand RNAs with transcripts of adjacent paralogous pseudogenes (2 cases) (i.e., *trans*-acting). Further examinations of five additional small RNA libraries revealed that pseudogene-derived antisense siRNAs could be produced in specific rice developmental stages or physiological growth conditions, suggesting their potentially important roles in normal rice development. In summary, our results show that pseudogenes derived from protein-coding genes are prevalent in the rice genome, and a subset of them are strong candidates for producing small RNAs with novel regulatory roles. Our findings suggest that pseudogenes of exapted functions may be a phenomenon ubiquitous in eukaryotic organisms.

## Author Summary

Pseudogenes are “defunct” copies of protein-coding genes that have been accumulated in a genome. They have conventionally been considered the junk byproducts of genome evolution, as they cannot code for proteins due to sequence degeneration. Recent important studies, however, have discovered that a subset of them have unforeseen roles as sources of non-coding RNA transcripts that can regulate the expression of functional coding genes. In this work, we have explored this perspective by studying to what extent rice pseudogenes can encode small RNAs, especially the antisense type. After cross-examining thousands of rice pseudogenes and several millions of small RNA sequences, we found that pseudogene-derived small RNAs are abundant in rice, and furthermore, many of them may function as natural antisense RNAs by interacting complementarily (based on sequence similarity) with sense RNAs from either parental genes or other pseudogenes. Some of these pseudogene RNAs may also be involved in repressing pseudogene transcription. Our findings shed new light on the multifaceted biological roles of pseudogene-derived siRNAs that may or may not affect their functional counterparts directly, indicating that more studies are required to explore in molecular detail the diverse functions of siRNAs originating from pseudogenes in both animals and plants.

## Introduction

Pseudogenes are genomic sequences derived from functional genes, but are often considered non-functional due to the accumulation of various deleterious mutations over their evolutionary history [1]–[6]. Compared with its parental gene (more precisely, the direct descendent of the ancestral gene that gave rise to the pseudogene), a pseudogene generally contains sequence features such as premature stop codon or frameshift mutations, due to relaxation of or entirely lack of functional constraints. Two major classes of eukaryotic pseudogenes have been described: processed and duplicated. Processed pseudogenes arose from retrotransposition events, i.e., the insertions of DNA materials into a genome via RNA intermediates. Duplicated pseudogenes, on the other hand, originated from DNA duplications. As a result, duplicated pseudogenes often retain the exon-intron structures of their parent genes, a characteristic absent in processed pseudogenes [1],[2],[4],[6].

Genome-wide pseudogene annotations have been carried out for several mammals and prokaryotes but to date not for rice or any other plants [3], [5], [7]–[11]. While previous studies have demonstrated that retrotransposition is the major mechanism for generating mammalian pseudogenes [3],[4],[6], it remains to be established whether duplication or retrotransposition is the predominant mechanism in plants such as rice.

Rice (*Oryza sativa*) is a very important crop species that supports about one half of the human population. The genomes of two sub-species (*indica* and *japonica*) were sequenced completely in 2005 [12],[13]. Based on current annotation (TIGR V5), the rice (*japonica*) genome contains 41,046 protein coding genes (excluding transposable elements, TEs), 763 tRNA genes, and 2,859 novel genes seemingly unique to rice and other cereal. To this date, no systematic and comprehensive annotation of pseudogenes has been done to address their impact on the structure and the function of the rice genome. However, a number of rice duplicated pseudogenes have been reported lately, including several arising from MADS-box genes, which encode a large family of transcription factors [7], and 99 pseudogenes in Cyt P450 family [8]. Moreover, current annotation has identified 15,232 TE-related genes and retrotranspositions have been documented to play a significant role in shaping the rice genome [14], suggesting that pseudogenes is a significant component of the rice genome.

While pseudogenes are usually considered non-functional, many transcriptionally active pseudogenes and several pseudogenes with exapted functions had been identified experimentally or suggested [2], [6], [9]–[11]. Generally, pseudogenes cannot be transcribed due to the lack of a functional promoter and auxiliary regulatory elements [1],[15]. However, a previous study revealed that at least a fifth of human pseudogenes could be transcribed to different degrees based on a variety of empirical transcription evidence, such as 5′ RACE (Rapid Amplification of cDNA Ends), tiling microarray analysis and high throughput sequencing data [5]. Other studies have also independently estimated that 5–20% of human pseudogenes exhibit evidence of transcription [16]–[19]. Likewise, a significant percentage of mouse pseudogenes were also found to produce stable RNA transcripts following the analysis of 100,000 mouse full-length cDNA [20]. The biological and functional implications of such pseudogene transcripts are largely unexplored, but a few of them have been indicated to play important biological roles mostly in gene regulation [1],[2],[6],[21],[22]. Moreover, direct evidence has been established for a functional NOS (nitric oxide synthase) pseudogene that is transcribed in specific neurons in the central nervous system of *L. stagnalis*, where its transcript forms a RNA duplex with the mRNA of its parental gene and curtains the production of NOS proteins [22]–[24]. The human XIST non-coding gene, the key initiator of X chromosome inactivation, also arose from the relic of a pseudogene [25].

Recent studies from deep sequencing of small RNA libraries have provided further strong evidence that a significant number of pseudogenes may serve as a genomic reservoir for functional innovation, e.g., as the source of small regulatory RNAs [9]–[11],[26]. Three major classes of small RNAs have been described in plants and animals: microRNAs (miRNAs), small interfering RNAs (siRNAs), and Piwi-interacting RNAs (piRNAs) [10],[27],[28]. MiRNAs are usually 20 to 24 nt long and they interact with targeted mRNAs to modulate their translations [27]. siRNAs, usually 21-nt long, are generated from double-stranded RNA precursors such as those from viruses or endogenous transposons [28]. In plants, five major groups of siRNAs have been reported: transacting siRNAs (ta-siRNAs), natural antisense transcript-derived siRNAs (nat-siRNAs), repeat-associated siRNAs (ra-siRNAs), heterochromatic siRNAs, and long siRNAs (lsi-RNA) [29]–[36]. In animals, piRNAs derived from repetitive elements via a Dicer-independent pathway have also been shown to repress the activity of mobile genetic elements [9], [37]–[39]. Meanwhile, siRNAs derived from long hairpin RNAs (hp-RNAs) can also repress endogenous target transcripts in *Drosophila* [40]. Most recently, it was shown that siRNAs originated from pseudogenes can regulate gene expression in mouse oocytes [9],[26]. More importantly, it was found that some of these pseudogene siRNAs were processed through Dicer and the loss of Dicer significantly reduced the number of pseudogene siRNAs, which in turn led to the up-regulation of their targeted genes [9],[26].

In plants, however, a plant-specific siRNA biogenesis pathway has been suggested to be responsible for the production and function of pseudogene-derived siRNAs. It was shown that the production of siRNAs from *Arabidopsis* pseudogenes depended on RNA-dependent RNA polymerase (RDR2) and Dicer-like protein 3 (DCL3) [41]. With a characteristic feature of 24-nt length, these pseudogene-derived siRNAs were thought to repress local transcription in a similar manner as the *cis*-acting siRNAs originated from transposons or retroelements [41], which can mediate RNA-directed DNA methylation (RdDM) and heterochromatin formation [42]–[47]. While this hypothesis is wholly consistent with the significant findings from the *Arabidopsis* work [41] and warrants more detailed investigations, it is not immediately clear how pseudogenes, especially those arising from gene duplications, can sustain DRD2 and DCL3 activities that seem to work most efficiently on tandem repeating sequences [42].

Motivated by these recent advances, we have carried out a genome-wide annotation of candidate pseudogenes in rice (*japonica* genome) and then carefully interrogated these pseudogenes for their capability of generating siRNAs. We annotated a total of 11,956 pseudogenes using a recently developed and validated pseudogene annotation pipeline, PseudoPipe [48]. Characterization of these pseudogenes with a library of ∼1.5 million small RNAs from developing rice grains [35] identified 145 pseudogenes as strong candidate loci for producing antisense small RNAs, with many of them having the potential to regulate the expression of their parental genes. Further survey of additional small RNA libraries indicated that the production of specific pseudogene-derived siRNAs could be restricted to particular developmental stages or growth conditions.

## Results

### Pseudogenes are prevalent in the rice genome

We have assigned a total of 11,956 pseudogenes, most of which contained premature stop codons and frameshift mutations (Table 1), in the rice genome using the PseudoPipe software [48]. Of the 41,046 non-TE-related protein-coding genes used as our query sequences, 4,946 (12.1%) had at least one pseudogene. Respectively, 3,392 and 2,350 of the rice pseudogenes were classified as processed pseudogenes and duplicated pseudogenes by PseudoPipe (Table 1). The rest (6,214) were designated as pseudogene fragments, since they did not contain sequence features that were considered by PseudoPipe to distinguish between retrotranspositions and DNA duplications. Such fragments are usually derived from duplications, so together with duplicated pseudogenes they are referred as non-processed pseudogenes. The average nucleotide sequence identity between pseudogenes and their parents was 70.5%, 76.6%, and 74.0% for processed pseudogenes, duplicated pseudogenes, and pseudogene fragments, respectively. The mean alignment coverage on the parental genes was 87.9%, 59.6%, and 35.2% for processed, duplicated, and fragments, respectively, suggesting that most past retrotranspositions generated rice processed pseudogenes of full length (in relation to the CDS of their parental genes). Detailed information for individual pseudogenes can be found in the annotation file available on the web (http://www.pseudogene.org/rice09/).

**Table 1:** Summary of rice non-RE-related pseudogenes annotated by the program PseudoPipe.

Pseudogene Type	Numbers of Pseudogenes	Numbers of Parents	Pseudogene Length (bp)	Sequence Identify (%) between Pseudogenes and Parents	Pseudogene Coverage (%) on Parents	Number of Stop Codons in Pseudogenes	Number of Frameshifts in Pseudogenes
Duplicated	2350	1672	748.3 (±619.3)	76.6 (±15.1)	59.6 (±27.0)	1.2 (±1.9)	1.7 (±1.9)
Processed	3392	1343	452.3 (±330.6)	70.5 (±14.0)	87.9 (±10.4)	1.3 (±1.6)	1.7 (±1.7)
Fragment	6214	2580	282.2 (±195.4)	74.0 (±14.4)	35.2 (±18.0)	0.9 (±1.3)	1.0 (±1.4)

**Figure 1:**
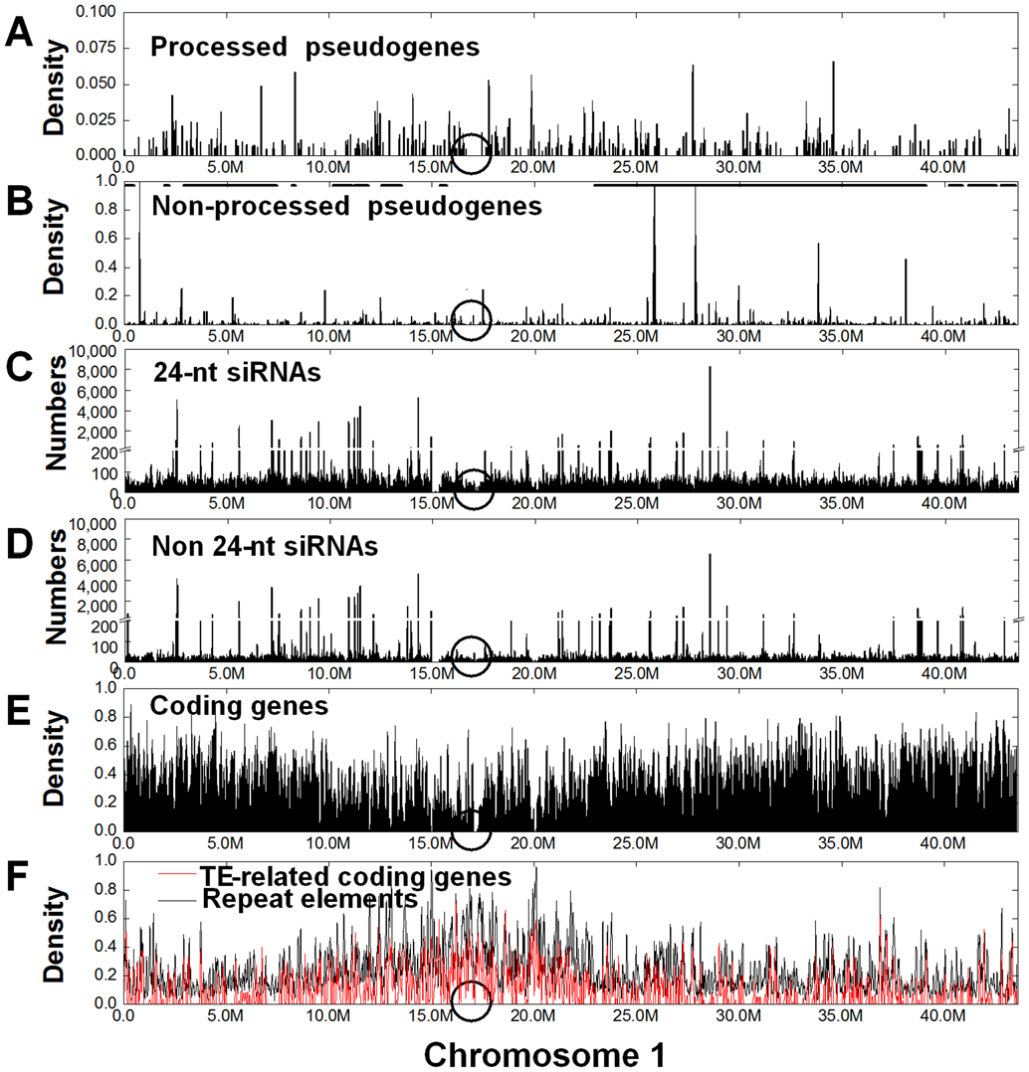
Genome-wide distribution of rice pseudogenes, siRNAs from developing rice grains, and repeats. Data shown here is for chromosome 1 only, but data for all chromosomes can be found in Supplementary Figure S1 and our website. Moving windows (50,000-nt length and 10,000-nt increment) were employed to calculate the proportion of nucleotides covered by processed pseudogenes (A), non-processed pseudogenes (B), non-TE coding genes (E), annotated TE coding genes or repeats annotated by the RepeatMasker program (F), or the number of siRNAs (C, D) in each window. The black lines at the top of B illustrate whole genome duplication regions and the open ovals mark the centromere.

Overall, the processed pseudogenes are randomly distributed in the rice genome, a pattern apparently different from the genome-wide distribution of non-processed pseudogenes (Figure 1 and Supplementary Figure S1). Moreover, non-processed pseudogenes are prevalently located in duplicated regions as expected from well documented extensive past duplication events in the rice genome. For example, a comparison of our pseudogene annotation with the whole genome duplication (WGD) data, described in [49], showed that 6,071 (70.9%) of rice non-processed pseudogenes are located in WGD regions, verse 2,085 (61.5%) of processed pseudogenes, in consistent with their distinct generation mechanisms (p = 1.88e−8).

**Figure 2:**
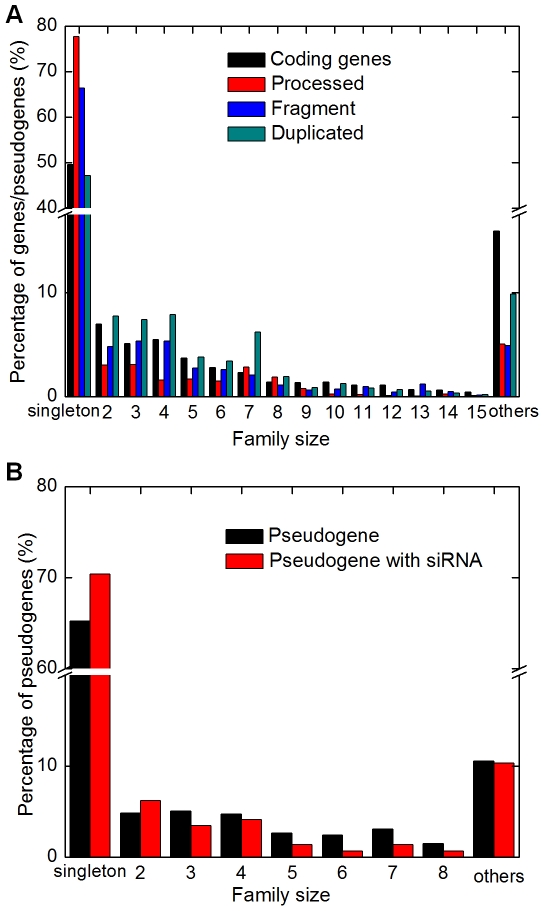
Percentages of genes/pseudogenes from gene families of different sizes. Genes vs pseudogenes are shown in A and total pseudogenes vs pseudogenes with significant numbers of antisense RNAs (group A in Figure 3) in B.

It has been reported that most of the rice genes can not be clustered into gene families [12],[13],[50], i.e., they do not have a functional rice paralog and thus are considered singleton genes. This is somehow counterintuitive with respect to the past active DNA duplications in rice. To investigate how this has impacted pseudogene biogenesis, we transferred the family annotation of each parental gene to its pseudogene(s), using gene family annotation from TIGR v5. Interestingly, this analysis showed that more than 65% of pseudogenes (*vs* ∼50% of coding genes) were from singleton gene families (Figure 2), and conversely 13.3% of singleton genes (*vs* 12.1% for all rice genes) had at least one pseudogene. These numbers imply that rice pseudogenes preferentially come from singleton families. Upon more careful investigation, however, we found that this bias was introduced largely by the specific method employed in annotating rice gene family, which clustered genes based on domain architectures of their protein products [50]. As a consequence, two genes sharing all but one domain would have been assigned to distinct families. We therefore utilized an alternative method that is better suited for our purpose in designating singleton genes: a gene is considered singleton if it does not share significant sequence similarity (BLAST e-value <1e-14) with other rice genes. By this criterion we found that only 7.6% (1,262) of singleton genes had a corresponding pseudogene, indicating that coding genes in singleton families are less likely to have a detectable pseudogene. Nevertheless, our results indicate that many singleton genes actually have a pseudogene relative even though they do not have a functional paralog. In conjunction with the TIGR rice gene annotation, our analysis suggests that the overabundance of singletons in rice genome is the outcome of sequence loss rather than sequence degeneration (i.e., pseudogenization).

Overall, we found that family size was negatively correlated with the number of pseudogenes in a family (R = −0.93, p<1e-15; Spearman's correlation computed for family size of 2 to 15), suggesting that large gene families do not necessarily have more “dead” (pseudogene) members (Figure 2), an interesting observation to be further studied.

The top ten genes with the most pseudogenes are LOC_Os01g10030 (550 pseudogenes), LOC_Os09g23670.1 (226), LOC_Os10g41910.1 (125), LOC_Os03g32980.1 (113), LOC_Os11g14500.1 (90), LOC_Os06g11360.1 (90), LOC_Os11g10210.1 (89), LOC_Os11g36210.1 (79), LOC_Os08g39680.1 (73), and LOC_Os01g59540.1 (67). The functions for eight of them have not been annotated but two appear to be housekeeping genes. This is strikingly different from what has been reported in mammalian genomes, where a large fraction of pseudogenes are derived from known gene families such as ribosomal protein genes and olfactory receptor genes [3],[5]. More specifically, the ribosomal protein genes have generated about 2,000 pseudogenes in both humans and mice [51] but only 50 in rice based on our current study. As the significant enrichment of processed pseudogenes from housekeeping genes in mammals is considered to be relevant to their high expression levels, current finding suggests that distinct evolutionary events are probably responsible for the generation and subsequent retainment of pseudogene populations in plants and animals.

### Some pseudogenes produce small RNAs in developing rice grains

**Figure 3:**
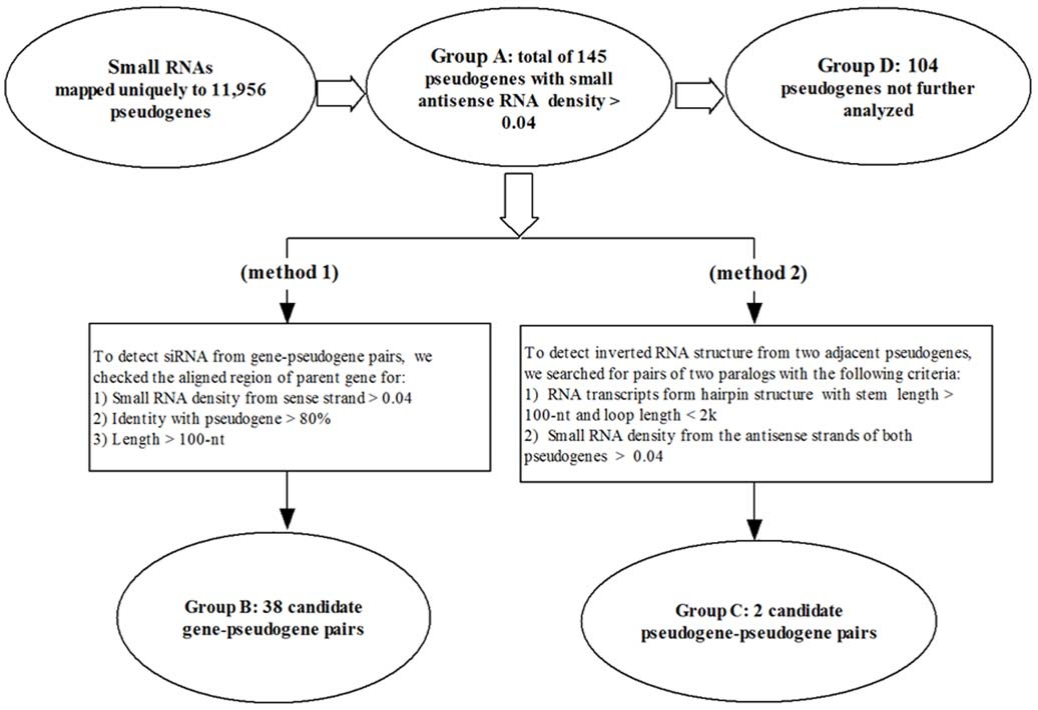
Overall workflow for identifying small RNAs derived from gene-pseudogene pairs or adjacent pseudogene-pseudogene pairs.

Recent studies have revealed that pseudogenes are an important source of non-coding RNAs [6],[9],[26]; furthermore, genome-wide small RNA analyses have indicated that pseudogene-originated siRNAs could either regulate the expression of their parental genes in mice [9],[26] or be implicated in silencing pseudogenes themselves in *Arabidopsis* [41]. To explore this, we have searched a library of small RNAs from developing rice grains [35] for antisense RNAs from pseudogene loci. Such RNAs would be complementary to the mRNAs from parental genes and thus may function as nat-siRNAs. We found that 2,867 and 2,582 pseudogenes had at least one small RNA mapped to their sense and antisense strands, respectively. By a threshold of >4 small RNAs/100 nucleotides (corresponding to ∼1× coverage, see Materials and Methods for details), we considered 145 pseudogenes as good candidates for producing antisense small RNAs (Figure 3, group A). Three-quarters (75.9%) of these candidates were from singleton families, in consistent with their abundant representation in the rice pseudogene population (Figure 2B). Additionally, 24 (16.6%), 39 (26.9%) and 82 (56.5%) of these pseudogenes were processed, duplicated and fragments, respectively, indicating a slight bias (p = 0.2) of non-processed pseudogenes in generating antisense small RNAs. For the convenience of description, we will use small RNAs and siRNAs inter-changeably below even thought the functions of the small RNAs in our dataset have not been established experimentally.

### Some rice pseudogene siRNAs show an RDR2/DCL3-dependent feature

**Figure 4:**
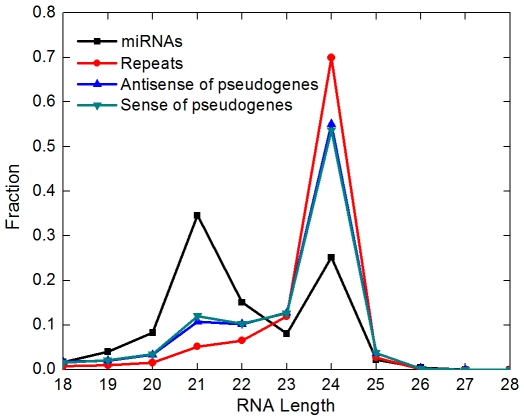
The length distribution of small RNAs from four distinct sources.

The sizes of antisense siRNAs, however, suggest that most of the rice pseudogene-derived siRNAs may not serve as nat-siRNAs, as the majority (53.4%) of them are 24-nt long (Figure 4), which is a common signature feature of small RNAs derived from plant RDR2 pathway. Our finding is rather consistent with what has recently been shown for pseudogene-derived siRNAs in *Arabidopsis* that were predominantly 24-nt long and depended on RDR2 and DCL3 for their accumulation [41]. The potential involvement of RDR2/DCL3 pathway is further supported by the observations that the majority of the 145 pseudogenes also produced some sense small RNAs and that the size distributions of sense and antisense small RNAs were identical (Figure 4). All together, these data indicate that ∼50% of pseudogene siRNAs in rice grains could have been produced by the Pol IV/RDR2/Pol V pathway. As plant 24-nt siRNAs and the Pol IV/RDR2/Pol V pathway are specifically implicated in RNA-directed DNA methylation (RdDM) and heterochromatin formation, two processes important for silencing transposons and other retroelements in plants [42]–[47], these results suggest that some pseudogene siRNAs may be important for *cis*-repression (more precisely, local-repression) of pseudogene transcription.

### Many rice pseudogene siRNAs show features distinct from ra-siRNAs

More careful analysis of small RNA sizes suggested that pseudogene-derived siRNAs might have other regulatory roles besides *cis*-acting repression in rice. It is known that trans-acting siRNAs (tasiRNAs) or nat-siRNAs in plants exhibit a size range quite different from that of repeat-associated siRNAs. For example, a recent analysis of rice nat-siRNAs found that their lengths varied from 17- to 31-nt with a detectable peak in 21-nt [36]. Interestingly, small RNAs from rice miRNA loci also displayed two peaks, at 21 nt and at 24 nt (Figure 4). In contrast, small RNAs from repeats (LINE, SINE, LTR and DNA transposons) exhibited a single strong peak at 24 nt (Figure 4). Therefore, we decided to study 24-nt and non 24-nt small RNAs separately, with the assumption that these two groups are generated from different pathways and have largely distinct functions. A comparison of the genome-wide distributions of these two groups of small RNAs, to our surprise, did not find an enrichment of 24-nt siRNAs in the centromeric and pericentromeric regions where repetitive elements are concentrated (Figure 1). These data accumulatively suggest that the repeat-relevant RDR2 pathway is not necessarily the sole pathway contributing 24-nt siRNAs to our small RNA library, and that pseudogene-derived siRNAs may have functions other than inducing local heterochromatin formation. It is worth to mention here that the first example of *trans*-acting nat-siRNAs, formed between SRO5 and P5CDH transcripts through the action of RDR6/DCL2 in *Arabidopsis*, is indeed 24-nt long [31].

### Complementary small RNAs from rice pseudogenes and their parents

In the studies of siRNAs in mice [9],[26], it was considered that simultaneous accumulation of sense-strand small RNAs from parental genes and antisense-strand siRNAs from pseudogenes in the complementary region(s) is good evidence for the production and as well as regulatory potential of pseudogene-derived siRNAs. Following this idea, we have searched for parental gene-pseudogene pairs in which both the parental genes and pseudogenes can produce significant numbers of sense and antisense small RNAs, respectively (see Materials and Methods for details). The complementary interaction of those RNAs can potentially affect the expression of the parental genes (or pseudogenes). Application of this strategy has yielded 38 gene-pseudogene pairs (Figure 3, group B) with the capability of producing *trans*-nat-siRNAs in developing rice grains. The over representation of non-processed pseudogenes (32 out of 38) lends a good support to our hypothesis, since small RNAs of *trans*-regulatory function arising from duplicated pseudogenes have previously been documented [6],[41],[52]. Again, one caveat here is that many of these 38 group B pseudogenes produced both sense and antisense siRNAs with a mixture of 24 and non-24 nt (Table 2). Based on sign-test statistical analysis, we found that 8 of the 38 group B pseudogenes (vs 16 of the 145 group A) had significantly more non-24 than 24 antisense siRNAs (at the false discovery rate of 5%), indicating that a RDR2 independent pathway is very likely involved. As an extra line of evidence for the existence of regulatory pseudogene siRNAs in rice grains, we found that none of the parental genes of the 38 group B pseudogenes had a matching EST from either rice seeds or seedlings, whereas seven and zero of the 145 group A pseudogenes had ESTs from seeds and seedlings, respectively.

**Table 2:** A list of 38 gene-pseudogene pairs with significant numbers of siRNAs.

Pseudogene							Parent gene					
Index	Sense RNA density	Antisense RNA density	Chr	S	Start	End	Locus ID	Sense RNA density	Antisense RNA density	S	Start	End
NP1*	0.407692	0.853846	chr10	−	2389454	2389713	LOC_Os10g04950.1	0.693487	1.065134	−	2397031	2397291
NP2*	1.134396	0.993166	chr1	+	28539222	28540984	LOC_Os03g51890.1	0.087083	0.050798	−	29699425	29705870
P3*	0.217842	0.357884	chr6	+	25789845	25790808	LOC_Os06g42910.1	0.137821	0.20406	+	25785529	25786464
NP4*	0.207143	0.667857	chr6	+	25788248	25788807	LOC_Os06g42910.1	0.161527	0.276065	+	25785787	25786467
NP5*	0.358559	0.657658	chr6	+	25786912	25787466	LOC_Os06g42910.1	0.134948	0.245675	+	25785779	25786356
NP6*	2.151767	1.432432	chr11	−	7728126	7729525	LOC_Os06g45920.1	0.229508	0.156648	+	27800192	27801148
NP7*	0.380362	0.590086	chr2	+	6776991	6778171	LOC_Os02g12840.1	0.38	0.590476	+	6776020	6778171
NP8*	0.076712	0.052055	chr9	−	13621436	13621899	LOC_Os09g22000.1	0.061625	0.019608	+	13323949	13324524
P9	0.408219	0.350685	chr3	−	29703341	29703705	LOC_Os01g16060.1	0.655462	0.736695	−	9038646	9039002
P10	1.025237	0.930599	chr1	−	9037869	9038185	LOC_Os01g16060.1	0.65	0.730556	−	9038646	9039005
NP11	0.102985	0.104478	chr3	−	12953347	12954016	LOC_Os03g22560.1	0.047904	0.02994	−	12941829	12943280
NP12	0.16637	0.068505	chr7	−	6773226	6775194	LOC_Os07g12130.1	0.22549	0.104278	−	6777805	6779755
NP13	0.152778	0.5	chr7	−	28194811	28195026	LOC_Os07g47160.1	0.186147	0.138528	−	28192801	28193031
P14	0.183511	0.180851	chr8	−	21292786	21293537	LOC_Os08g34180.1	0.335019	0.240202	−	21292212	21296576
NP15	0.417373	0.226695	chr8	−	21295511	21295982	LOC_Os08g34180.1	0.373239	0.239437	−	21294721	21296589
NP16	0.306316	0.155789	chr8	−	10887045	10888077	LOC_Os08g35680.1	0.431414	0.425131	−	22373656	22376218
P17	0.175055	0.135667	chr10	−	2388369	2388825	LOC_Os10g04940.1	0.502415	0.42029	−	2396072	2396574
NP18	**0.918919**	**0.880309**	**chr10**	**−**	**2394179**	**2394437**	**LOC_Os10g04950.1**	**0.693487**	**1.065134**	**−**	**2397031**	**2397291**
NP19	0.051756	0.072089	chr12	+	1260526	1261066	LOC_Os11g09160.1	0.142593	0.101852	−	4892269	4895253
NP20	0.084142	0.064725	chr12	−	10181112	10181420	LOC_Os12g17670.1	0.190939	0.116505	−	10117389	10117697
NP21	0.518519	0.462963	chr12	−	10176790	10176951	LOC_Os12g17790.1	0.141975	0.228395	−	10180695	10180856
NP22	0.201058	0.259259	chr12	−	10120640	10120828	LOC_Os12g17790.1	0.125	0.180556	−	10180695	10182331
NP23	0.247312	0.521505	chr12	−	10116988	10117173	LOC_Os12g17790.1	0.134409	0.209677	−	10180695	10182301
NP24	0.421196	0.119565	chr7	−	4995089	4995778	LOC_Os01g17990.1	0.125333	0.08	+	10061176	10061877
NP25	0.189451	0.187298	chr1	+	32638886	32640039	LOC_Os01g56110.1	0.051282	0.051282	+	32630547	32633482
NP26	0.296636	0.136086	chr2	+	6767726	6776915	LOC_Os02g12840.1	0.579505	0.299028	+	6774030	6777908
NP27	0.136134	0.112605	chr2	+	12224482	12225076	LOC_Os02g43460.1	0.047945	0.025685	+	26231834	26232417
NP28	0.066482	0.146814	chr5	+	7173611	7174240	LOC_Os05g12510.1	0.086022	0.124731	+	7172070	7174240
NP29	0.064516	0.059423	chr5	+	7171726	7172825	LOC_Os05g12510.1	0.073684	0.101754	+	7172079	7174354
NP30	0.056911	0.178862	chr5	+	7171459	7171581	LOC_Os05g12510.1	0.089431	0.113821	+	7172331	7174159
NP31	0.694051	0.300283	chr5	+	25269356	25270679	LOC_Os05g43582.1	0.108656	0.042357	+	25264352	25265642
NP32	0.348649	0.421622	chr7	−	21686946	21687465	LOC_Os07g36300.1	0.122616	0.125341	+	21707670	21711406
P33	0.233463	0.215953	chr8	+	813356	813869	LOC_Os08g02280.1	0.093407	0.076923	+	815516	816061
NP34	0.147436	0.269231	chr8	+	17590709	17591464	LOC_Os08g28940.1	0.113782	0.123397	+	17568707	17570466
NP35	0.245232	0.256131	chr8	+	17574263	17576763	LOC_Os08g28940.1	0.159307	0.218182	+	17566446	17570788
NP36	0.366667	0.193333	chr9	+	6548157	6548396	LOC_Os09g11590.1	0.070968	0.006452	+	6469198	6469532
NP37	0.924647	0.362637	chr9	+	16537008	16537858	LOC_Os09g27190.1	0.43349	0.184664	+	16532458	16533770
NP38	0.183168	0.15099	chr10	−	2399336	2399739	LOC_Os10g04890.1	0.073563	0.098851	+	2359638	2360072

**Figure 5:**
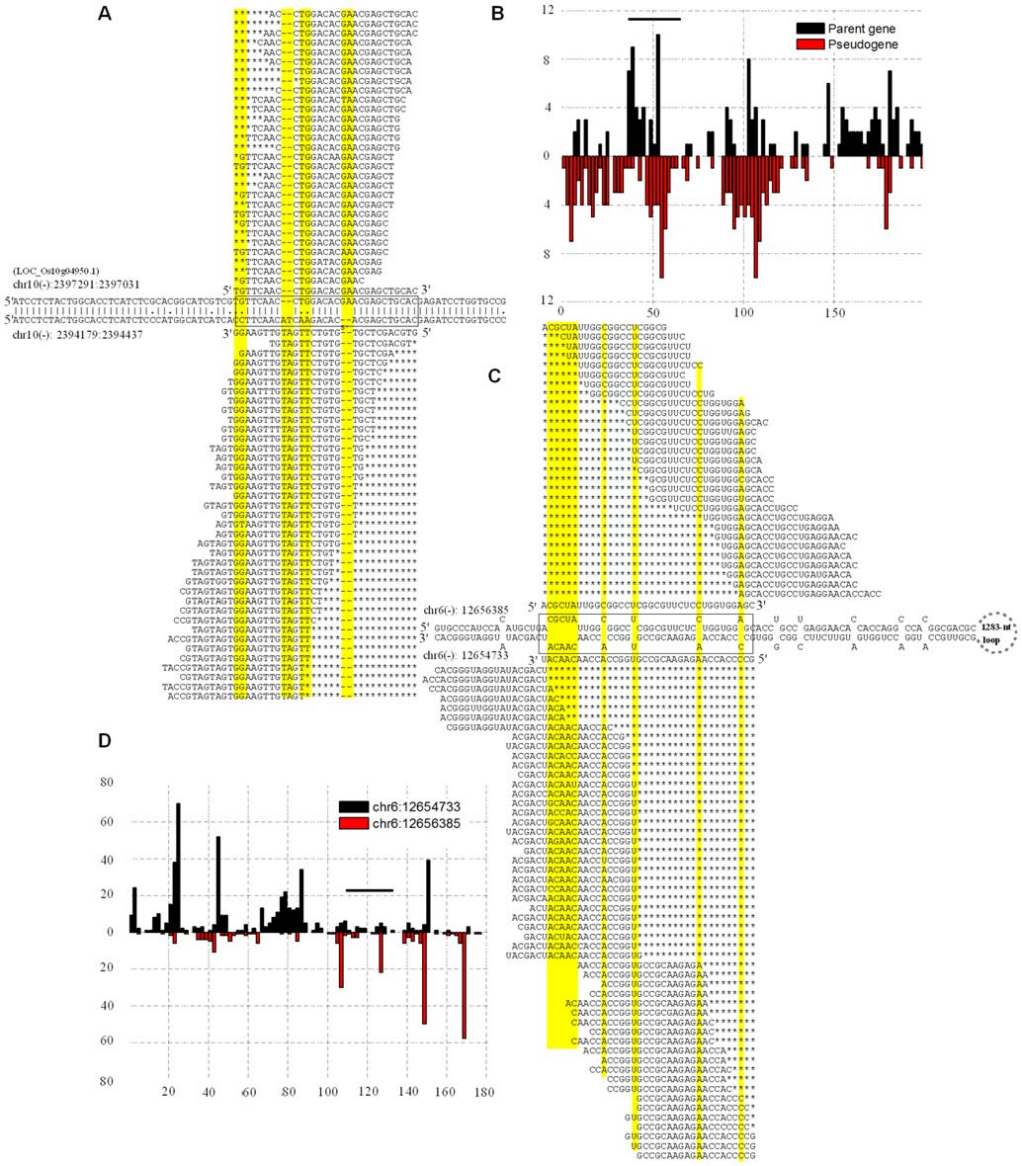
Examples of regulatory small RNAs derived from pseudogenes. One (A, B) is for gene-pseudogene interaction and the other (C, D) is from a pair of inverted pseudogenes forming predicted RNA duplex. The numbers of small RNAs mapped unambiguously to the top strand (in black) and the bottom strand (in red) are shown in B and D, and the counts are for small RNAs with their starting positions located in a 2-bp bin. Data in the top and bottom of A and B are for the gene (sense) and its pseudogene (antisense), respectively, while the data in the top and bottom of C and D are for a pseudogene and its paralogous pseudogene on the opposite strand. Unique small RNAs from the boxed regions are listed in A and C with yellow columns highlighting unique sites or indels for placing siRNAs specifically to top or bottom sequences.

In the index column, “P” is for processed and “NP” for non-processed pseudogenes. NP18 (bold entry) is illustrated with details in Figure 5 (A, B). “S” indicates the strand directions of genes or pseudogene. Pseudogenes with significantly more non-24 than 24-nt antisense siRNAs are marked by a “*”.

Intriguingly, the pseudogene and its parental gene in 21 of these 38 pairs in group B (55.3%) are very close to each other on chromosomes (<10 kb) (Table 2). By comparison, we found that only 437 of our 11,956 pseudogenes (3.7%) were within 10 kb of their parental genes. These data indicate that siRNAs with a potential of forming (or from) nat-RNA duplex are significantly more likely to be generated from gene-pseudogene pairs of short distances (χ^2^ = 171.0, p<1e-15). It should be mentioned here that none of these 21 pairs are located in the previously identified siRNA generation hotspots [53]. As an example, one pair defined by the gene LOC_Os10g04950 and its pseudogene 2,594-bp downstream contained large numbers of unique small RNAs exclusively mapped (based on polymorphism) to the sense strand of gene or the antisense strand of pseudogenes (Figure 5, A and B). In this case, we also noticed some antisense RNAs from the parent gene and sense RNAs from the pseudogene. The importance and implication of their presence need to be explored in the future.

### Complementary small RNAs from adjacent paralogous pseudogenes

The discovery of nat-siRNAs from gene-pseudogene pairs prompted us to search for other types of mechanisms that could also be important for the production of functional pseudogene siRNAs. It has previously been shown that siRNAs can be derived from hairpin RNAs [9],[26],[40]. In the search of such small RNAs (Figure 3), we identified two pairs of pseudogenes that potentially could generate transcripts forming hairpin structures (Table 3). One example is a pseudogene located on chromosome 6 (from 12,656,179 to 12,656,385-bp) and its downstream paralogous pseudogene (12,654,917 to 12,654,733-bp; the parent of these two pseudogenes is LOC_Os02g46460). The RNA transcript(s) of these two pseudogenes was predicted to form a 185-nt duplex linked by a 1,283-nt loop (Figure 5). A large number of small RNAs were uniquely mapped to the inverted repeat duplex predominantly to the minus strand, indicating that the transcripts were produced from minus strand of the two pseudogene loci (Figure 5, C and D). We did not detect any small RNAs from the parental gene of these two pseudogenes.

**Table 3:** Two pairs of inverted pseudogenes whose transcripts can form RNA hairpin structures.

ID	Strand	Identity	5′ Start	3′ End	Length	Sense RNA Density	Antisense RNA Density
**chr6:12656179..12656385**	**+**	**83**	**12656201**	**12656385**	**185**	**0.08696**	**2.67633**
**Paralogous**	**−**		**12654917**	**12654733**	**185**	**1.56284**	**0.04372**
chr2:6767726..6776915	+	96	6776546	6776698	155	1.858	0.06452
Paralogous	−		6777870	6777717	154	0.0714	1.883

The first case is illustrated with details in Figure 5 (C, D).

In summary of our analysis of small RNAs from developing rice grains, a total of 145 pseudogenes were identified as good candidates for generating pseudogene-derived antisense endo-siRNAs, with the parental genes for 38 of them harboring significant number of sense siRNAs, suggesting that a small subset of rice pseudogenes might have evolved exapted functions to produce regulatory antisense small RNAs. Our finding extends the previous observations from mouse oocytes [9],[26] and *Arabidopsis* [41], and suggests that siRNA-modulated regulatory function of pseudogenes may be conserved from animals to plants.

### Conditional expression of pseudogene-derived siRNAs

**Figure 6:**
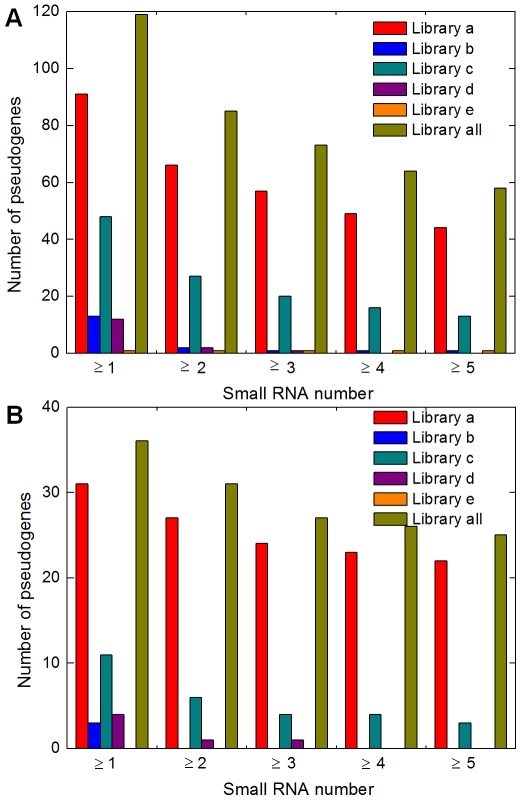
Numbers of pseudogenes with antisense small RNA in developing rice grains and five additional libraries. Here, A is for the 145 group A pseudogenes and B is for the 38 pseudogenes (group B in Figure 3) that produce siRNAs in developing rice grains. The x-axes represent the numbers of siRNAs from the other five libraries. Information for the libraries a–e is described below. Library all is the union of a to e. a. Dehulled mature grain, 141,370 small RNAs (matching uniquely to rice genome, ditto below). Heisel et al., 2008 [53]; b. 23 days old seedlings, 58,863 small RNAs. Heisel et al., 2008 [53]; c. A mixture of RNAs by MPSS from (1) seedlings treated with ABA, (2) nipponbare immature panicles - 90 days old plants, (3) germinating seedlings infected with Magnaporthe grisea, (4) germinating seedlings, (5) stem, and (6) seedling control for ABA treatment. 136,870 small RNAs. Nobuta et al., 2007 [54]; d. Four-week old seedlings, 73,174 small RNAs. Zhou et al., 2008 ; e. CRSDB, a mixture of RNAs isolated from 30 to 60 day leaves (∼16.5%), 10, 25 and 30 day seedlings (∼11%), 4–7 cm inflorescences (∼16.5%) and 25 day seedling polysomes (∼16.5%), 5,521 small RNAs. Johnson et al., 2006 [55].

We have further explored the diversity of pseudogene-derived siRNAs using additional five small RNA datasets acquired in different rice developmental stages and physiological growth conditions (details in Figure 6 and Supplementary Table S1). Obtained with different high-throughput sequencing techniques, these five datasets contained (a) 285,873 [53], (b) 108,472 [53], (c) 299,454 [54], (d) 182,792[36], and (e) 11,809 [55] small RNAs, approximately half of which in each dataset could be mapped to the rice genome uniquely (Figure 6). These new datasets are considerably smaller than our primary dataset described above, which had ∼1.5 million mapped RNAs, therefore we did not apply the threshold of 4 RNAs/100 nt to them. The numbers of pseudogenes with 1∼5 antisense small RNAs in these libraries and their comparison with our primary RNA data can be found in the Supplementary Table S1, indicating that 2,350 and 82 rice pseudogenes had ≥1 or ≥5 antisense small RNA in at least one of the five datasets, respectively. Moreover, 43.9% (1,134/2582) of the pseudogenes with ≥1 antisense siRNA(s) in our primary grain library were found to have siRNA(s) in the pool of these five new datasets. Among the 145 group A pseudogenes (Figure 3), 119 (82.1%) could be detected with ≥1 antisense small RNA, and more strictly, 58 (40%) with ≥5 antisense RNAs in at least one of the five datasets (Figure 6A, library all), strongly supporting the existence of endo-siRNAs from those pseudogenes. When considering the source of rice materials used for individual libraries, however, we found that most pseudogenes with antisense siRNAs from rice grains (≤10 days-after-fertilization) did not have matched siRNAs in other libraries, including those from more developed rice seedling. For example, 91 of the 145 (62.8%) group A pseudogenes were detected with antisense siRNAs from the dehulled grain library obtained by Heisel et al. (Figure 6A, library a), but only 13 of them (9.0%) had siRNAs in the library of 23 days old seedling (Figure 6A, library b). The difference here (p = 1.2e-6) is not simply the consequence of sequencing depth, as library a is no more than three times bigger than library b. Similarly, antisense siRNAs were also detected for most (31) of our 38 group B pseudogenes (Figure 3) in the Heisel's rice grain dataset (a) but not (only 5) in non grain-related datasets (b, d, e) (Figure 6B). Such skewness is even more pronounced when we examined pseudogenes with more than one siRNA in the new libraries (Figure 6). These comparisons of small RNA libraries from a variety of sources showed that many pseudogenes seemed to produce antisense siRNAs only under specific developmental stages or physiological conditions. Therefore, the pseudogene-derived siRNAs detected only in rice grains may play important roles in early rice grain development, a topic worthy of future investigation.

### GO functional categories of rice pseudogene siRNAs

**Figure 7:**
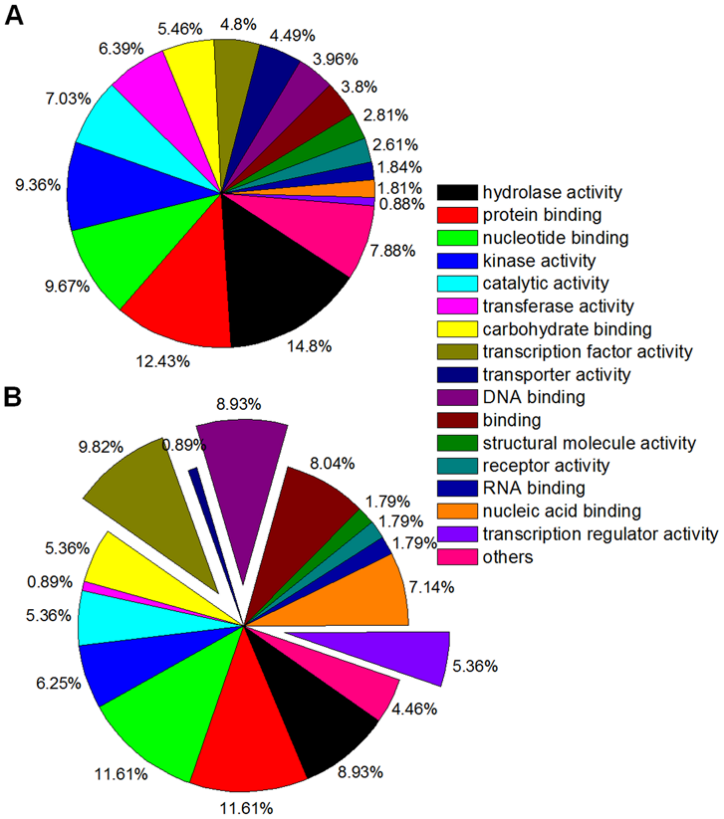
Function classification of rice pseudogenes. Panel A is for all pseudogenes and panel B is for group A pseudogenes (Figure 3) with antisense small RNAs in developing rice grains. The GO terms of a pseudogene were transferred from its parent gene with the functional categories defined by TIGR Gene Ontology (GO). A “*” indicates the significantly enriched GO in 145 pseudogenes with antisense small RNAs.

Our analysis of Gene Ontology (GO) showed that the majority of the parent genes with pseudogenes producing siRNAs in developing rice grains were implicated in gene regulation. For all rice pseudogenes, the most representative function was hydrolase activity (14.8%) (Figure 7A). This bias seems to be specific to plants, but consistent with previous observations for mammalian genomes housekeeping functions such as protein binding (12.4%) and nucleotide binding (9.7%) were also relatively abundant [3],[5]. However, it has to be cautioned that these statistics might have been complicated by that fact that 32% of 11,956 pseudogenes have not been assigned a GO functional category. In comparison to all rice pseudogenes, we found that the 145 group A pseudogenes with many antisense siRNAs in developing rice grains were substantially enriched in GO terms implicated in DNA binding and transcription regulation (p<0.05) (Figure 7B). This result indicates that pseudogene-derived antisense siRNAs could affect more downstream targets in a broader content by regulating the expression of the parental genes.

## Discussion

Pseudogenes are conventionally viewed as the by-products of genome evolution. About one-fifth of the rice protein coding genes are transposable elements [14], and the continuous activity of TEs has generated numerous TE-related pseudogenes (∼26,000 identified in this study), a feature common to many plant genomes. It is also known that gene duplications have occurred quite frequently in the rice genome [56]–[58]. For example, it was estimated that 15% to 62% of the rice genome underwent a whole genome duplication (WGD) ∼70 million years ago [56],[57]. A more recent segmental duplication event has also left ∼3 Mb synteny between chromosome 11 and 12 [58],[59]. Our observation of a high percentage of non-processed pseudogenes in the rice genome, and particularly their relative enrichment in the WGD region over processed pseudogenes, is consistent with these previous reports. On the other hand, at least 35% of the rice genome is considered the product of retrotransposition events mediated by retrotransposons such as *copia* and *gypsy* elements [13],[60]. That estimation is in line with the percentage (28.4%) of processed pseudogenes annotated in current analysis. However, in comparison to the human or rodent genomes, in which about half of their pseudogenes are derived from retrotranspositions, rice has much fewer processed pseudogenes. Moreover, the rice genome has a lower pseudogene to gene ratio (12000 *vs* 41,000) than the mammalian genomes (approximately 20,000 *vs* 22,000) [51]. It will be interesting to study in the future how much this is related to the rice genome's compact size and rapid sequence loss after retrotranspositions or gene duplications [57]. Another avenue to explore is whether many of the predicted rice genes without experimental evidence are actually pseudogenes, an important concern not addressed in current work. Although our pseudogene annotation is largely consistent with the known evolutionary history of the rice genome, the exact ratio of rice processed verse duplicated pseudogenes could deviate from the number reported here, since PseudoPipe [48],[61] was originally developed for mammalian genomes.

It is very intriguing that so many rice genes (∼50%) are presented themselves as singletons (TIGR v5) [50] even though duplications have been highly active during the evolution of the rice genome. In current work, we found that 13.3% of rice singleton genes have pseudogene relatives despite that the majority (1,729, 64.2%) of them have only one pseudogene. We had expected more singleton genes to have pseudogene relatives, based on high frequency of DNA duplications in rice and the reasonable assumption that most of the duplicated sequences associated with singleton genes should be detected as pseudogenes. The small percentage of singleton genes with pseudogenes (actually 7.6% based on a more strict definition of singleton family, see Results), in conjunction with the large number of singleton coding genes, indicated that most of the rice sequences resulted from past duplications have been either deleted or altered too substantially to be recognized by simple sequence comparison. This is in full accordance with the general view in the field that deletion and degeneration are the predominant outcome for duplicated sequences [62]–[64]. Moreover, an analysis of the synonymous substitution ratio (Ks) between pseudogenes and their parental genes showed that the non-processed pseudogenes from singletons (Ks = 0.26±0.39) appear younger than those from multi-gene families (0.46±0.61) (p<0.001), implying that the failure of detecting “old” pseudogenes from singletons might indeed be a reason. As a comparison, we found that 36.5% of *Arabidopsis* pseudogenes were generated from singleton genes and conversely 11.5% of *Arabidopsis* singleton genes had a pseudogene relative. These results suggest that domestication process could have introduced significant interference to the nature selection in rice. Nonetheless, there are rice singleton genes that have generated many pseudogenes. For example, the top four singleton genes (LOC_Os01g10030, LOC_Os08g13800, LOC_Os09g02850 and LOC_Os11g25600) with the most pseudogenes have generated a total of 717 pseudogenes, accounting for 6.0% of the rice pseudogenes. This is remarkable especially considering that so many (singleton) genes do not have a protein coding paralog but their pseudogene derivatives may produce antisense siRNAs. Our finding might also be relevant to the differences in functions and alternative splicing between singletons and multi-gene families [50].

While pseudogenes derived from functional genes have lost their protein coding potential, they may have gained novel biological functions. This topic has not received adequate attentions, but it is supported by the discovery of a functional NOS pseudogene in snails [22]–[24], and furthermore by recent studies that employed deep sequencing to show that a subset of pseudogenes in mouse oocytes can produce regulatory siRNAs [9],[26]. Our discovery of a large number of antisense siRNAs from rice pseudogenes and especially the finding of 38 parental gene-pseudogene pairs with many complementary small RNAs suggest that some rice pseudogenes could have evolved novel functions by encoding nat-siRNAs. To certain extent, our result is complementary to a recent finding that a large number of rice coding genes can produce *cis*-nat and *trans*-nat siRNAs, the majority of which also seem to be associated with specific growth conditions or developmental stages [36]. Alternatively, the pseudogene-derived siRNAs might not regulate functional genes but play a *cis*-acting role in recruiting RNAi machinery to suppress local transcription through the plant-specific Pol IV/RDR2/Pol V pathway [42]–[47]. The latter scenario, however, cannot explain completely the existence of sense siRNAs in parental genes. These two possibilities are not necessarily mutually exclusive. Firstly, siRNAs from processed pseudogenes can be involved in *cis*-acting as they are generated from retrotranspositions, while siRNAs from non-processed pseudogenes can function in *trans* similar to how some known miRNAs evolving from pseudogenes regulate their targets [6],[41],[52],[65]. Secondly, the same pseudogene might produce both *cis*-acting (presumably 24-nt) siRNAs and *trans*-acting (non 24-nt) siRNAs depending on how the pseudogene transcript is processed and utilized by RNAi machinery. Even in the case of *cis*-acting 24-nt siRNAs, their potential roles in regulating local gene expression should not be ignored. For example, RdDM and formation of repressive chromatins mediated by pseudogene siRNAs can affect the expression of a parental gene if it is sufficiently close to its pseudogene in the chromosomal space due to repression spreading. In our study, we also observed small RNAs derived from adjacent paralogous pseudogenes on opposite strands. Although these pseudogene siRNAs might be taken by the cellular siRNA machinery to modulate the expression of functional genes, they can also be important for suppressing pseudogene expression. It is conceivable that double stranded RNAs can form *in vivo* between pseudogene antisense siRNAs and their complementary sequences, but only carefully designed molecular and cellular experiments will resolve the different functional scenarios discussed here, for example, by identifying the specific Argonaute proteins and RNA-induced silencing complex (RISC) interacting with pseudogene siRNAs. As most of the studies on 24-nt repeat-associated siRNAs, RDR2/DCL3 and RdDM have been conducted in the model plant organism *Arabidopsis*, we hope our analysis and similar recent work [35],[36] will draw the interests of rice molecular biologists to study endo-siRNAs as there seems to be a difference in the association of 24-nt siRNAs with repetitive regions especially in the centromeric regions between rice and *Arabidopsis* genomes (Figure 1 here *vs* Figure 2 in ref 41).

In our current study, we have focused on pseudogenes that produce a significant number of antisense small RNAs. This does not mean that small RNAs from other pseudogene loci are spurious and less likely to be biologically relevant. Rather, the lack of support by multiple RNAs is largely technical, namely the insufficient depth of high-throughput sequencing. For example, 82–92% of the small RNAs from rice grains were only detected once in deep-sequencing using Illumina or 454 sequencing technology [35]. Therefore, low expression and inadequate sampling are probably the reasons for not finding more pseudogene siRNAs. These surely have added a caveat to our results in the comparison of different small RNA datasets with a variety of sequencing depths. On the other hand, pseudogene-derived small RNAs can be much more complicated than what is described here. For example, we found that two pseudogenes with high sequence similarity in very narrow complementary regions could also generate many siRNAs (data not shown). As suggested by previous investigators, such small RNAs may actually be an important source of novel miRNAs [6],[11].

Finally, we have also explored the potential regulators for the transcription of these pseudogenes derived endo-siRNAs. In particular, we have examined the genomic distance of pseudogenes to various transposable elements (included DNA transposon, LTR element, LINE and SINE). A total of 1,062 and 1,108 rice pseudogenes were found to be near (<1 kb) active transposable elements (defined as >90% of the full length TEs) in their 5′ sense and 3′ antisense directions, respectively (Table 4). DNA transposons were the most predominant TEs both in the 5′ sense (788 or 73.3%) and the 3′ antisense directions (811, 73.2%). The enrichments of these TEs in the flanking regions of pseudogenes are significant based on randomization simulations. However, only seven of the 145 pseudogenes with abundant antisense small RNAs have a TE oriented in the antisense direction in their 3′ flanking regions, suggesting that TE promoters are unlikely the primary driver for the production of endo-siRNAs from rice pseudogenes. The initiation of the transcription of these pseudogenes therefore remains perplexing regardless whether the primary transcripts can be subsequently used to produce *cis* or *trans*-acting siRNAs.

**Table 4:** Statistics for transposable elements in the flanking region of pseudogenes.

Pseudogene	S/A	LTR	SINE	LINE	DNA transposon
Duplicated	S	38	3	1	132
	A	44	5	0	121
Fragment	S	131	14	0	437
	A	137	16	1	462
Processed	S	82	13	2	209
	A	87	5	2	228
Total	S	251	30	3	778
	A	268	26	3	811
Permutation Average	S	107.46 (10.78)	11.07 (3.29)	0.76 (0.87)	387.23 (19.51)
	A	109.02 (9.93)	11.41 (3.36)	0.72 (0.82)	393.98 (19.24)
P-value	S	<0.001	<0.001	<0.01	<0.001
	A	<0.001	<0.001	<0.01	<0.001

“S” means 5′ sense direction of pseudogene and “A” means 3′ antisense direction. Standard deviation is shown within parentheses.

## Materials and Methods

### Data source

Rice genomic sequences and their annotations (release 5) were downloaded from the Rice Genome Annotation of TIGR (The Institute of Genomic Research, ftp://ftp.tigr.org). About 5.5 million small RNA sequences, generated by CSIRO (Commonwealth Scientific and Industrial Research Organization) for 1–5 days-after-fertilization (DAF) and 6–10 DAF rice grains using high throughput sequencing [35], were downloaded from the Gene Expression Omnibus (GEO) (GSE11014, http://www.ncbi.nlm.nih.gov/geo/). From this dataset a total of 1,483,951 small RNA sequences matching to rice genome uniquely (i.e, with a single best aligned location in up to one mismatch) were obtained as our primary RNA dataset. Five additional small RNA datasets were also obtained, including (a) 285,873 unique small RNAs from the tissue of rice grains assayed with three replicates of independent libraries and (b) 108,472 small RNAs from rice seedling downloaded from the GEO (GSE13152) [53], (c) 299,454 17-bp MPSS small RNA sequences from University of Delaware (http://mpss.udel.edu/rice/) [54], (d) 182,774 unique small RNAs matching perfectly to the rice genome collected from the library of control (58,781), drought (43,003) and salt (80,990) seedlings [36], and (e) 11,809 small RNA sequences downloaded from CRSDB (Cereal small RNA database, http://sundarlab.ucdavis.edu/smrnas/) [55]. Small RNAs from these five additional datasets were mapped to rice genome by the program BLAST [66]. The results were parsed by in-house scripts to extract the chromosomal coordinates of RNAs matching to rice genome uniquely with at most one mismatch in order to ensure comparability between data from our primary library and these additional new libraries. All subsequent analyses utilized genomic coordinates unless specified otherwise.

### Pseudogene analysis in the rice genome

For pseudogene assignment, we applied the PseudoPipe program [48],[61] to the rice genome with its repetitive sequences masked by the RepeatMasker (http://www.repeatmasker.org). Briefly, PseudoPipe scanned the rice genome for DNA sequences similar to a library of annotated rice protein sequences in the TIGR release V5. After those overlapping with annotated genes were discarded, the rest of matching DNA fragments was assembled into pseudogene candidates based on their structural similarities to the query proteins. Details of the pseudogene discovery procedure have been described previously [48],[61]. At the end, a total of 11, 956 pseudogenes derived from non-TE protein coding genes were identified in the rice genome. These pseudogenes were classified into duplicated pseudogenes, processed pseudogenes, and pseudogene fragments based on the potential mechanisms of their generations [3]–[5]. Thus, each of these pseudogenes was defined by its sequence and structural similarity to a functional gene (or protein), commonly referred to as the parental genes. Our pseudogene annotation is available publicly (http://www.pseudogene.org/rice09/). The sequence identity and coverage (the percentage of parental DNA material present in the alignment) between a pseudogene and its parent are part of the information in our annotations.

### Search gene-pseudogene pairs and inverted pseudogene paralogs for potential source of nat-siRNAs

The high sequence similarity between a pseudogene and its parent gene suggests that a natural *trans* RNA duplex can be formed between the antisense transcript from the pseudogene and the sense transcript from the parent gene. The cellular siRNA/RICS machinery can often use such double strand RNAs to produce mature and functional siRNAs. Here we focused our study on antisense small RNAs from pseudogenes by cross-referencing our pseudogene annotation with the library of small RNAs from ≤10 DAF rice grains, which has the largest collection of RNAs among all the publicly available datasets. As described above, we excluded small RNAs that were aligned to more than one location in the rice genome. For each pair of pseudogene and parent gene, their putative coding DNA sequences were first re-aligned using the global sequence alignment program NEEDLE in the EMBOSS package [67]. We then counted the numbers (N) of small RNAs mapped to the alignment region(s) to generate two numbers for genes, one for the sense and the other for the antisense strand, and likewise two corresponding numbers for pseudogenes. These numbers were then divided by the length of the alignment (L) to obtain the densities (N/L) of small RNAs for each pair of gene and pseudogene. We chose the density of 0.04 as a cutoff in order to select candidate pseudogenes with high potential to produce RNAs. This threshold is equivalent to ∼1× sequence coverage of small RNAs as the length of small RNAs is 18∼25 nt [35]. Furthermore, for a pair of parental gene and pseudogene to be considered as a strong candidate with the potential to produce nat-siRNAs, the sense RNAs of the gene and the antisense RNAs of the pseudogene must both meet the density cutoff. The overall protocol and the resulting different groups of pseudogenes are shown in Figure 3.

We also searched for cases in which the transcripts of two adjacent pseudogenes can form RNA hairpin structures. To do so, we used BLASTN program to search paralogs of each pseudogene with antisense RNAs. Paralogous pairs within 2 kb of each other but on opposite strands were evaluated for the potential of producing hairpin RNAs. We used two criteria to define pairs that could generate siRNAs from the RNA hairpin: 1) the stem of the hairpin >100-bp and the loop length <2 kb, and 2) the density of small RNAs (from the same strand) mapped exclusively to the inverted hairpin >0.04 (Figure 3).

### Statistical analysis

All statistical analyses were carried out in the R language. When characterizing all pseudogenes, we used the GO function categories of their parents and simply reported GO terms that were highly represented among rice pseudogenes without further inferring statistical significance. To evaluate the functional significance of the 145 pseudogenes enriched with antisense siRNAs (density >0.04 siRNA per nt), we considered the GO distribution of all pseudogenes as the genome-wide background and then employed a re-sampling approach to infer the enrichment of a specific GO term. We selected 145 pseudogenes randomly from all pseudogenes and then recorded the number of pseudogenes in each GO. After 10000 iterations, we obtained an empirical p-value for each GO term measuring the number of iterations that more than X pseudogenes was observed in a GO, where X was the number of pseudogenes in this GO from the 145 pseudogenes.

To identify transposable elements in close proximity of pseudogenes, we considered LTR, SINE, LINE and DNA transposons defined by RepeatMasker annotation with these two criteria: (1) distance to pseudogene is <1 kb and (2) annotated TE covers >90% of its consensus full length (thus considered as “active” TE). So, data discussed here are for pseudogenes located downstream (<1 kb) of an active TE. A re-sampling protocol was used to test the significance of a TE enrichment, which was done by choosing a random region with the same length of a pseudogene from the same chromosome that this pseudogene was located. A total of 11,956 such regions were selected and then the number of TEs within flanking regions of these randomly selected regions was counted. By repeating this process 1,000 times, we derived an empirical p-value for TE enrichments. We carried out this analysis for TEs oriented in the 5′ sense and 3′ antisense directions of pseudogenes separately.

## Supporting Information

Figure S1 Genome-wide distribution of rice pseudogenes, siRNAs from developing rice grains, and repeats.
(10.17 MB DOC)

Table S1 Comparison of pseudogenes with antisense siRNAs in different small RNA libraries.
(0.07 MB DOC)
