# Supplementary material for: Small RNAs Originated from Pseudogenes: cis- or trans-Acting?
Source: PLoS Comput Biol. 2009 Jul 31;5(7):e1000449. doi: 10.1371/journal.pcbi.1000449 (PMC2708354; doi:10.1371/journal.pcbi.1000449)

Small RNAs Originated From Pseudogenes, *cis*- or *trans*-Acting?

Xingyi Guo, Zhaolei Zhang, Mark B. Gerstein, and Deyou Zheng

**Figure S1.** Genome-wide distribution of rice pseudogenes, siRNAs from developing rice grains, and repeats. Moving windows (50,000-nt and 10,000-nt increment) were employed here to calculate the proportion of nucleotides covered by processed pseudogenes (A), non-processed pseudogenes (B), non-TE coding genes (E), annotated TE coding genes or repeats annotated by the RepeatMasker program (F), or the number of siRNAs (C, D) in each window. The black lines at the top of B illustrate whole genome duplication regions and the open ovals mark centromeres.


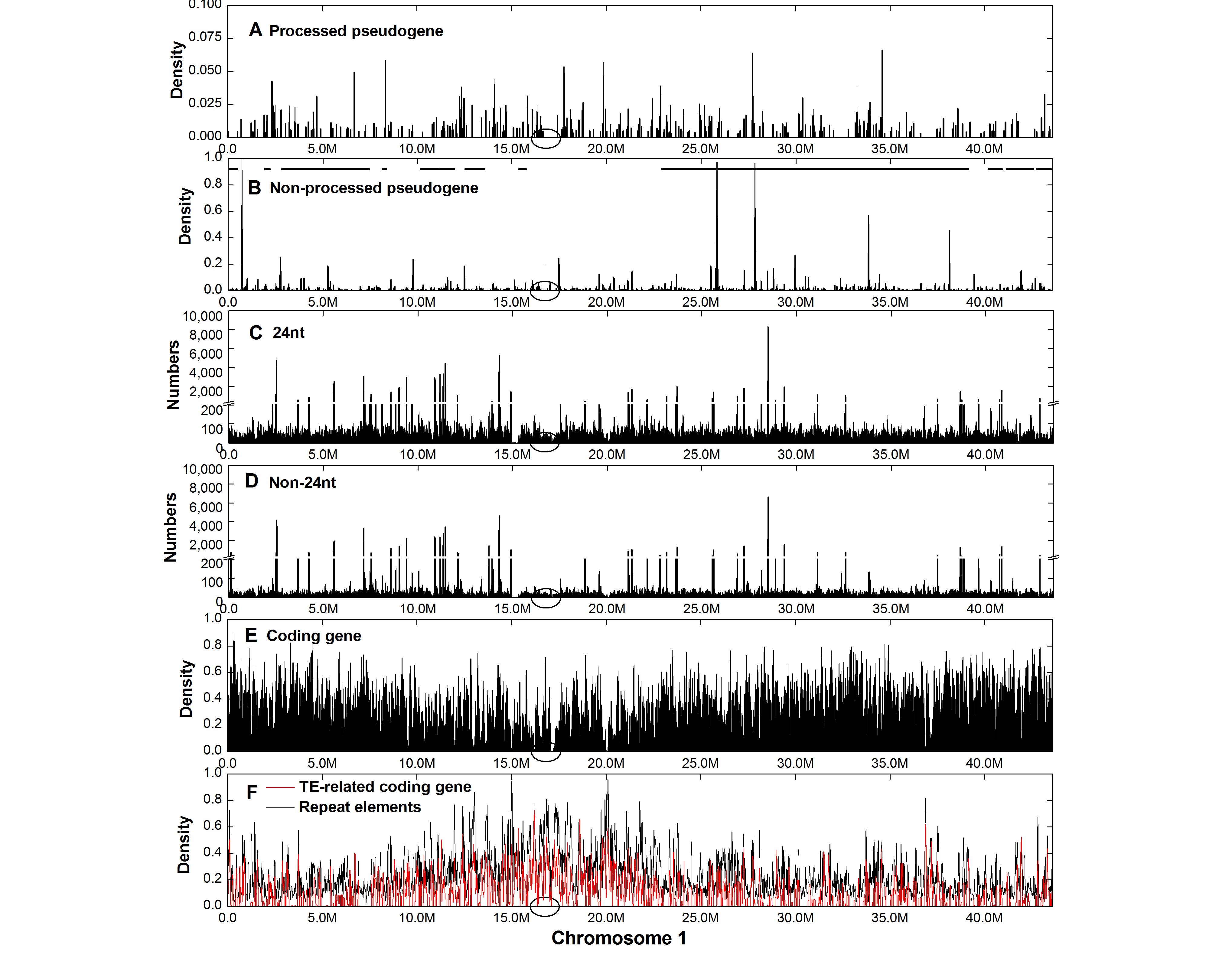


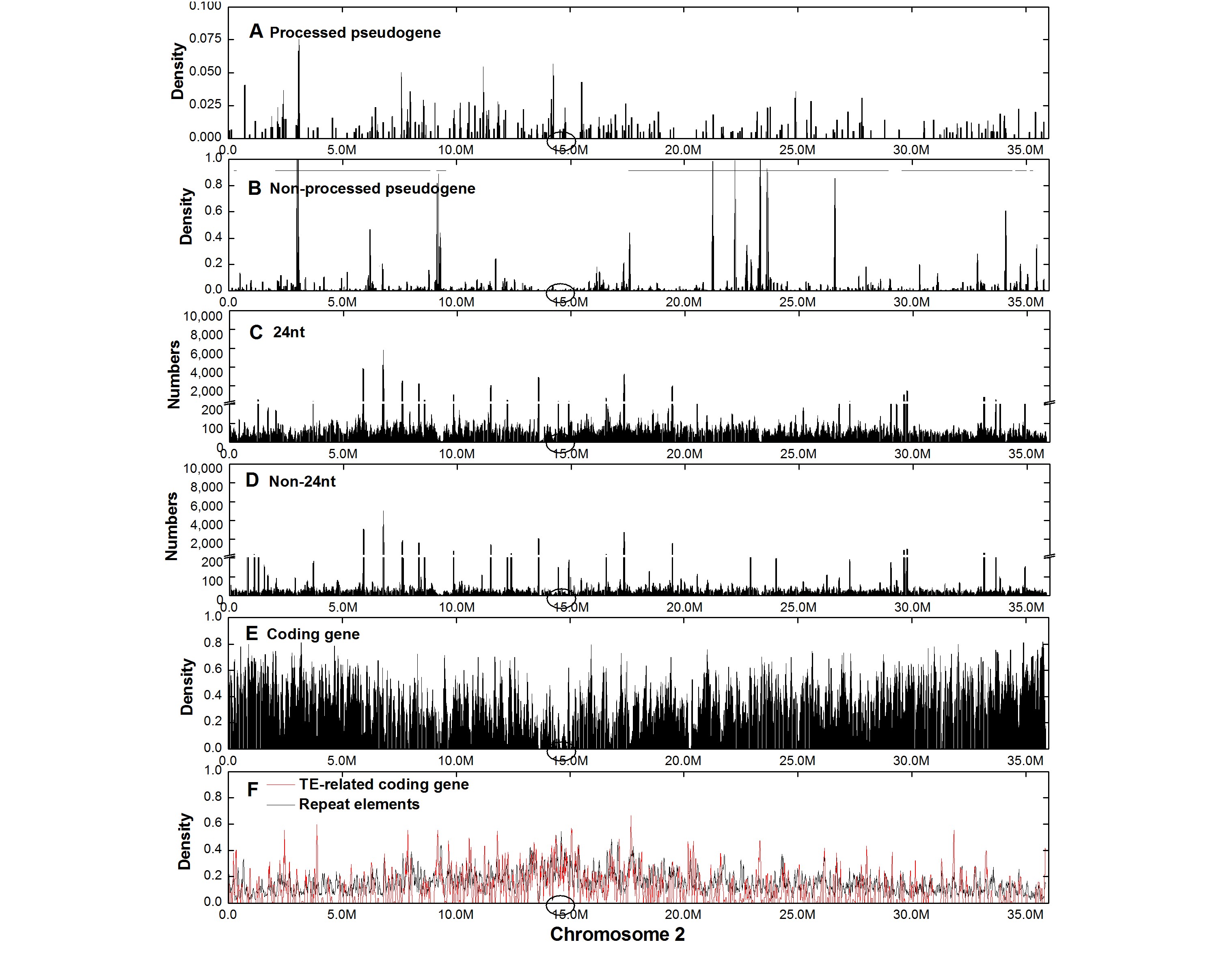


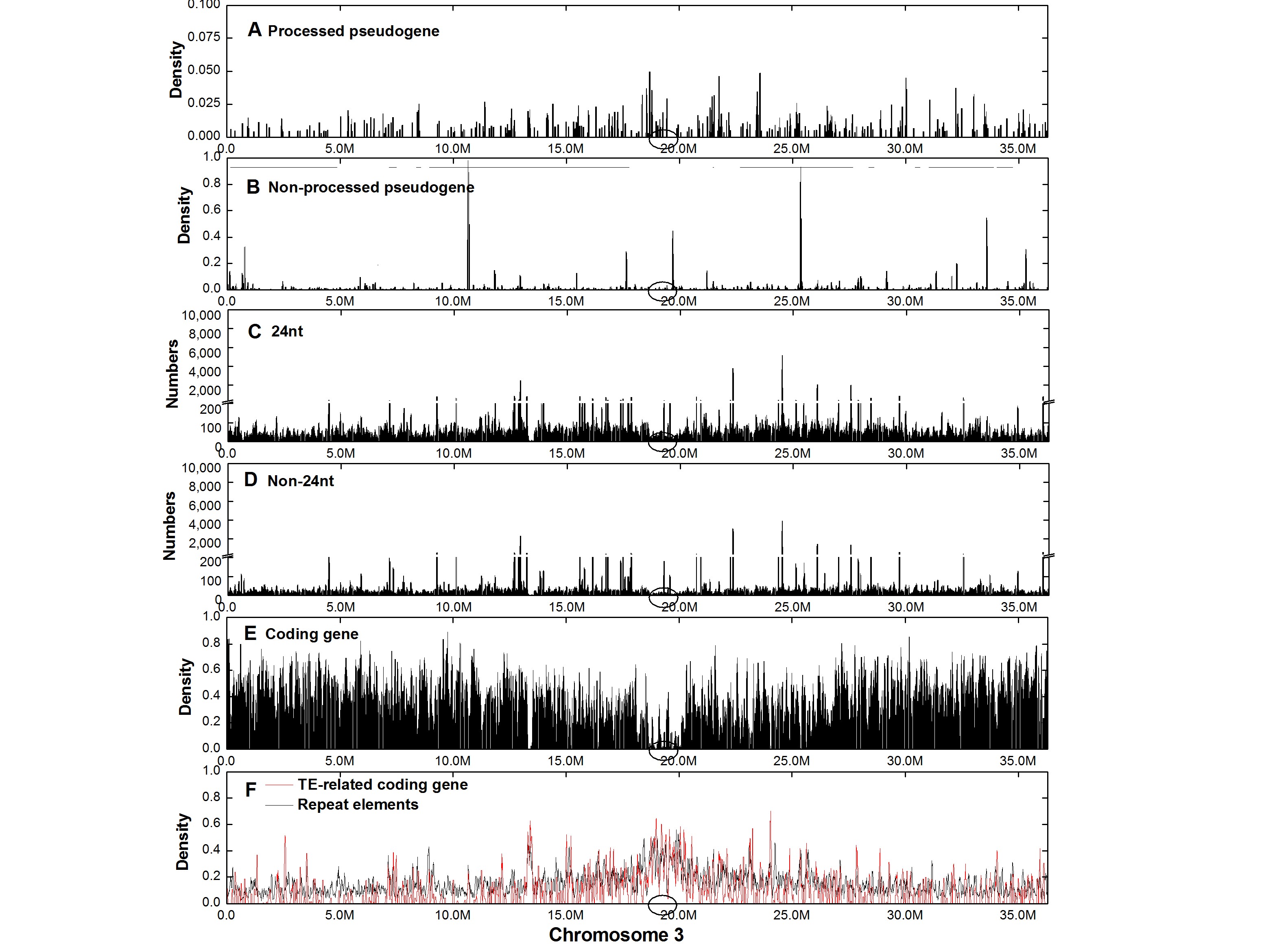


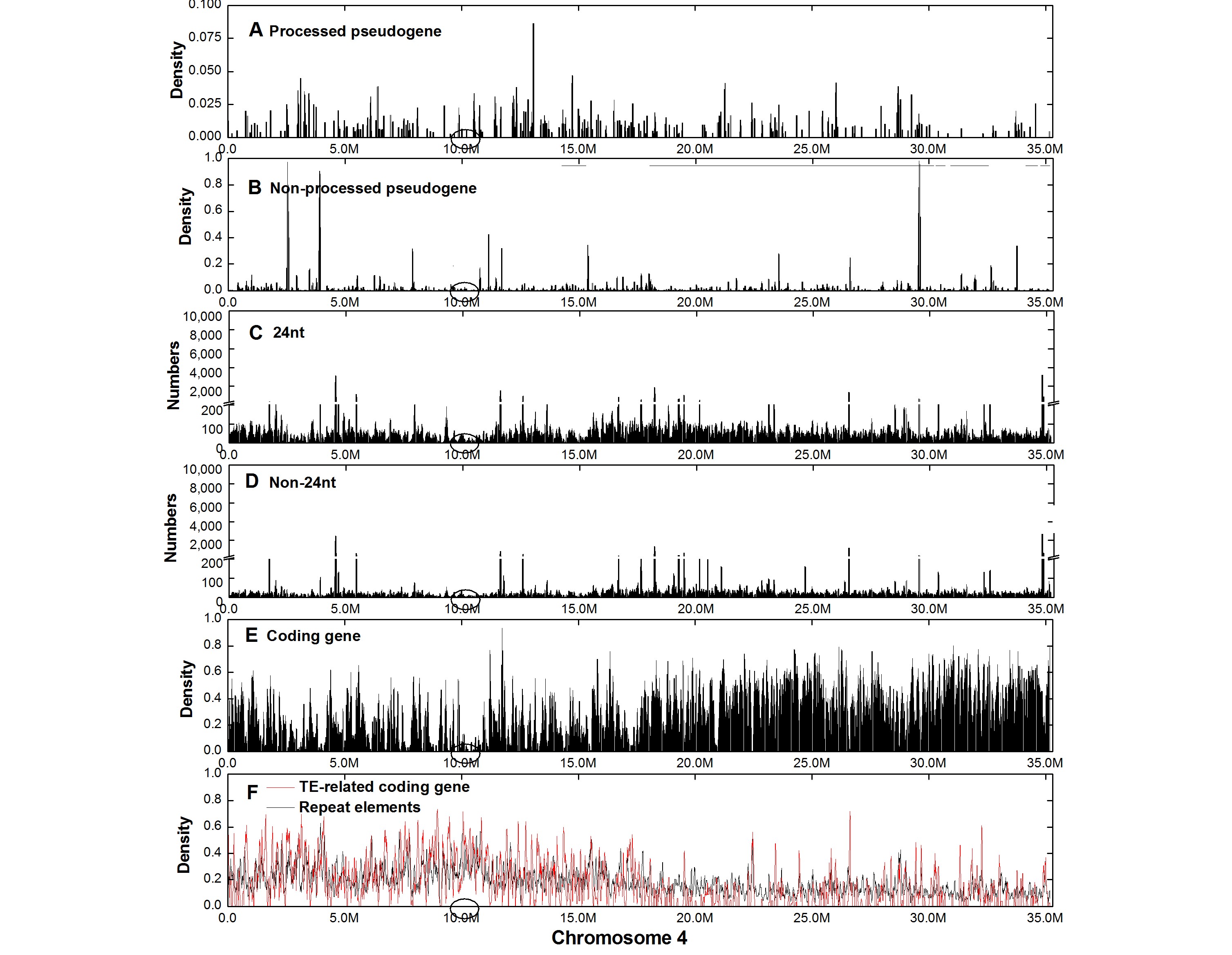


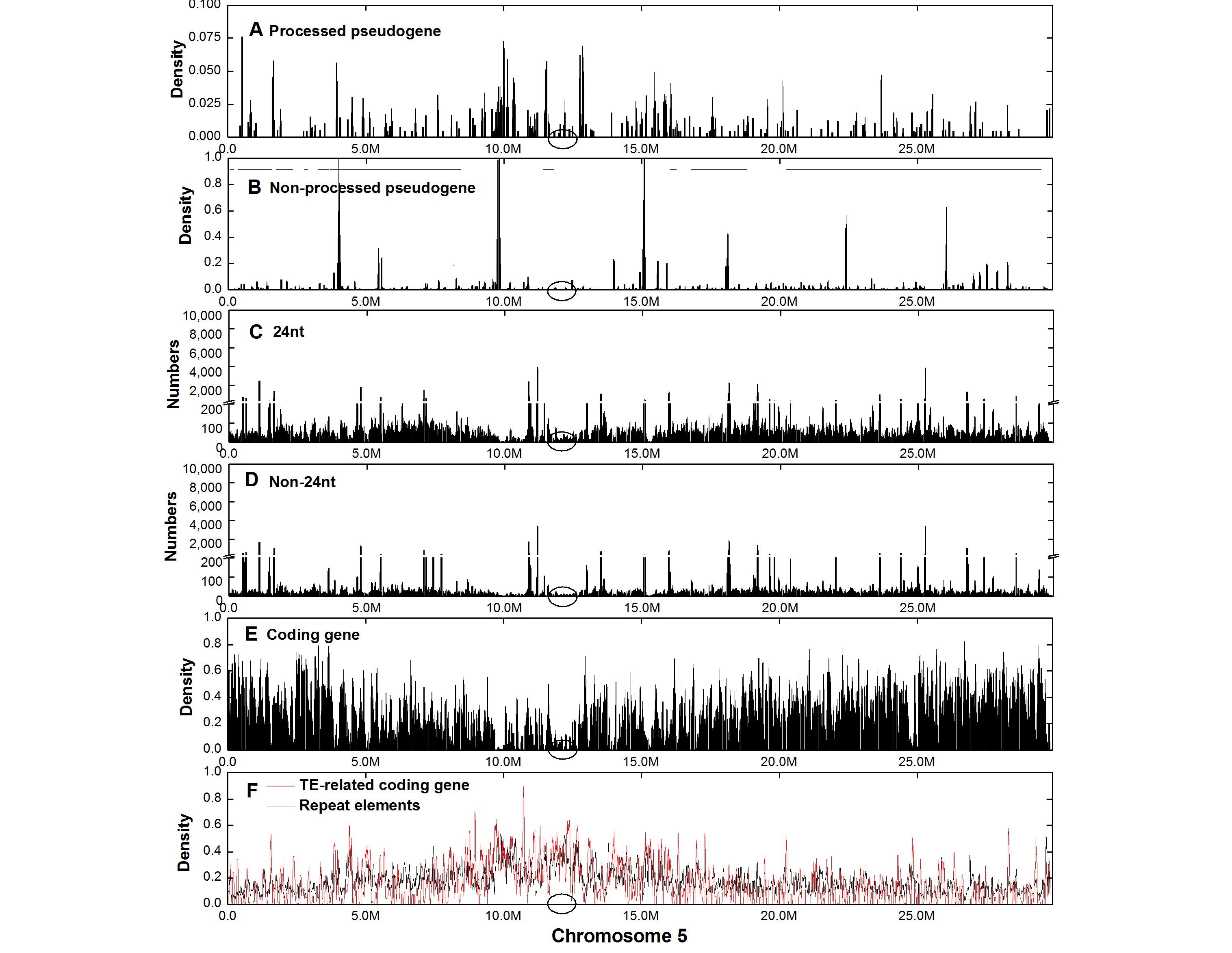


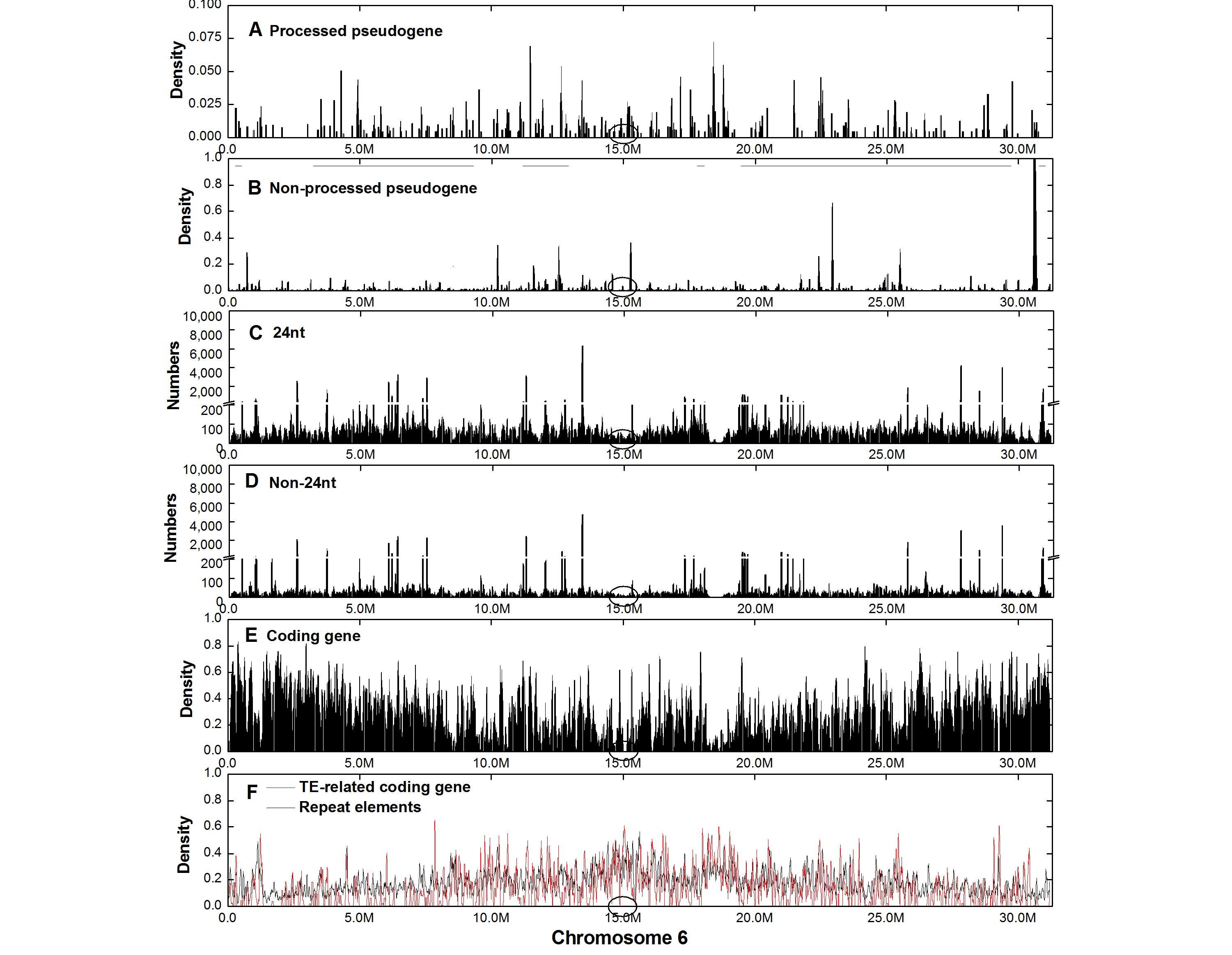


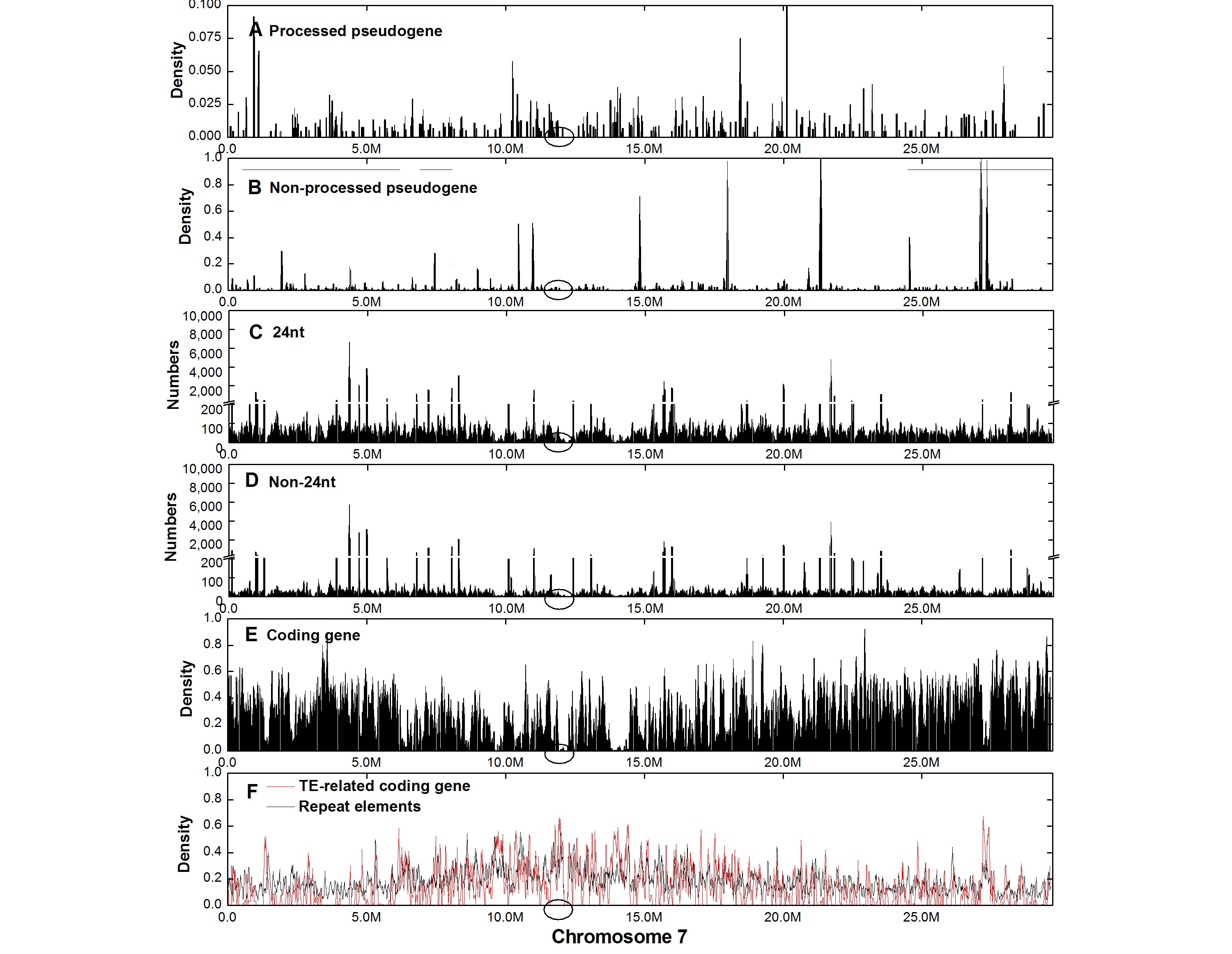


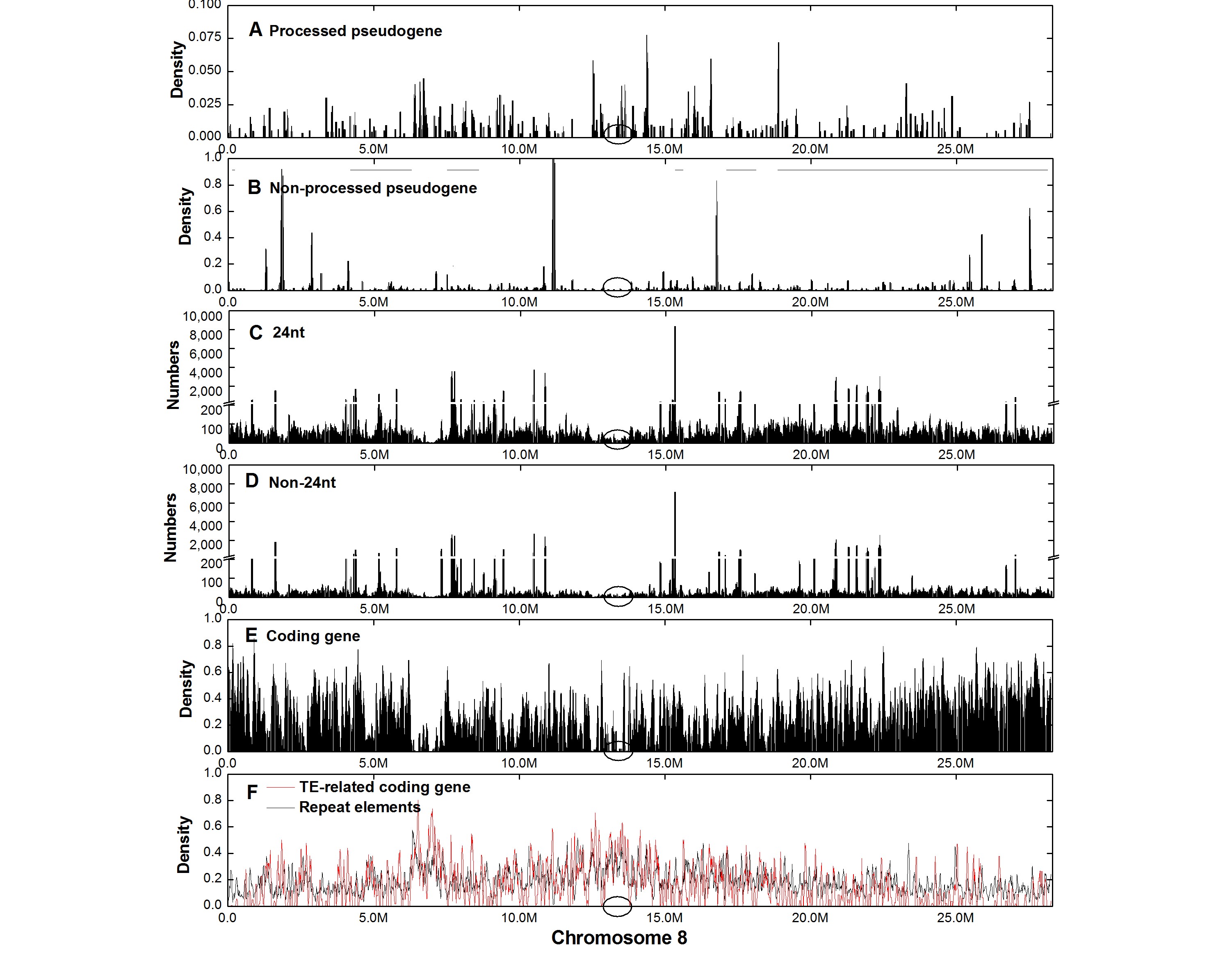


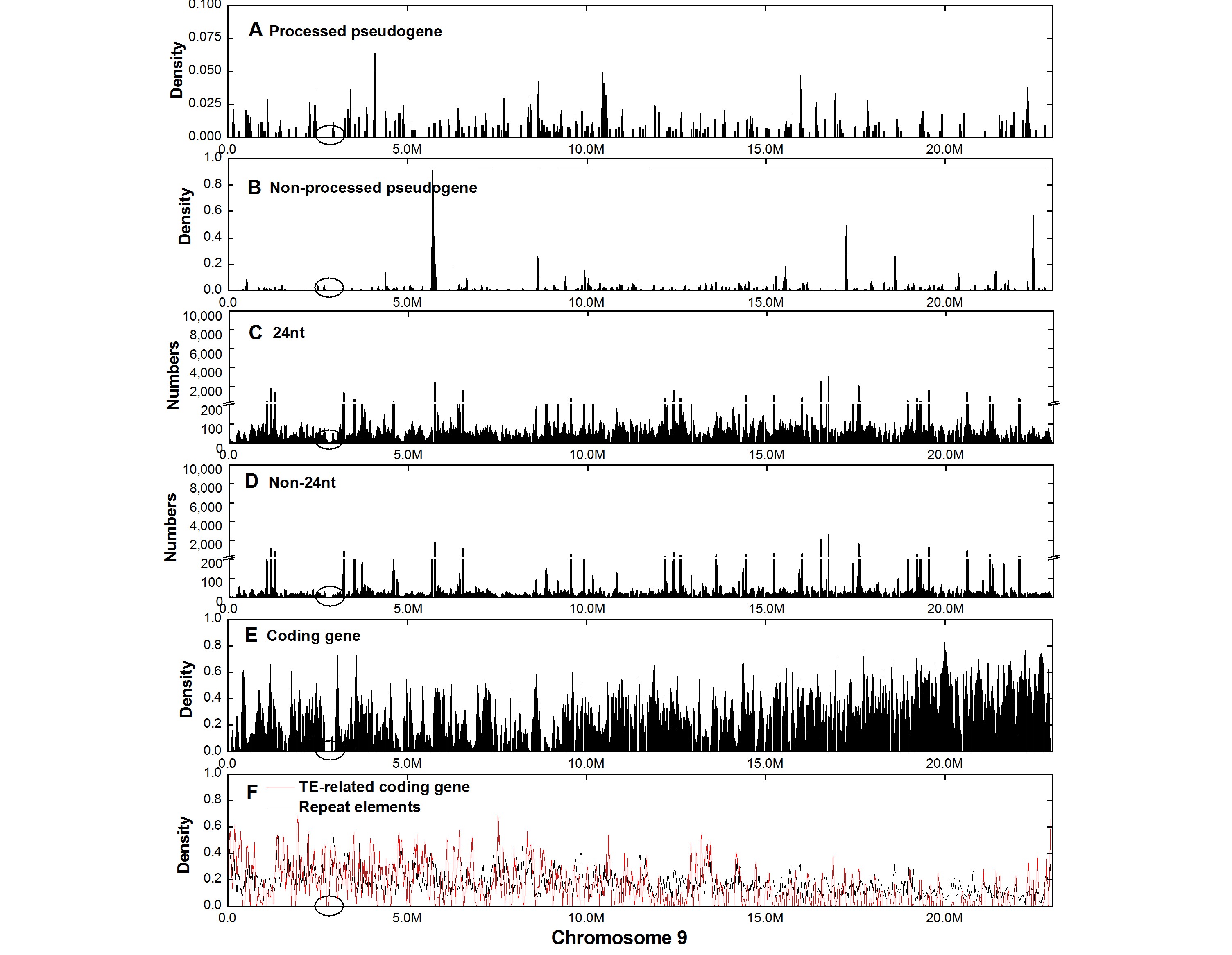


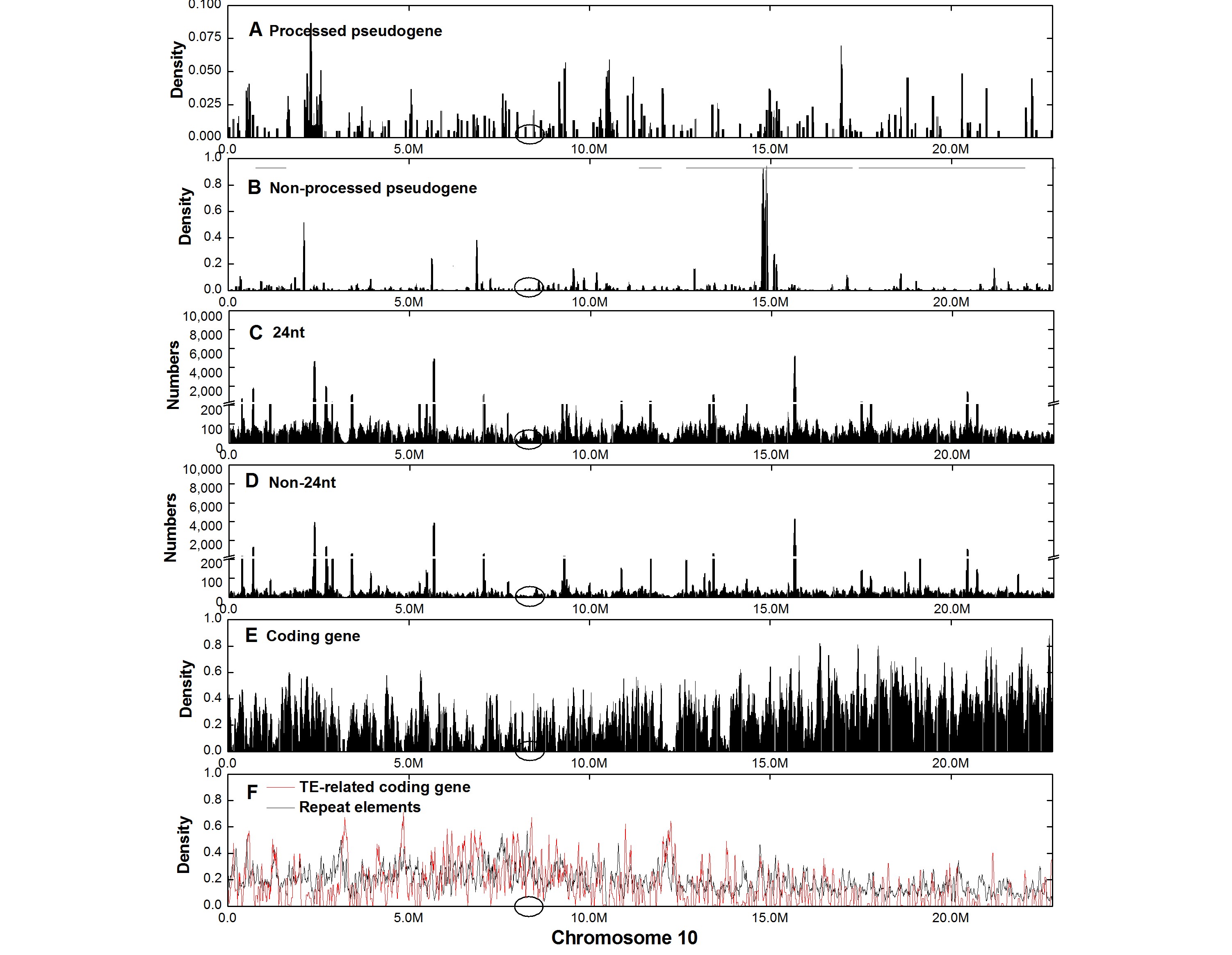


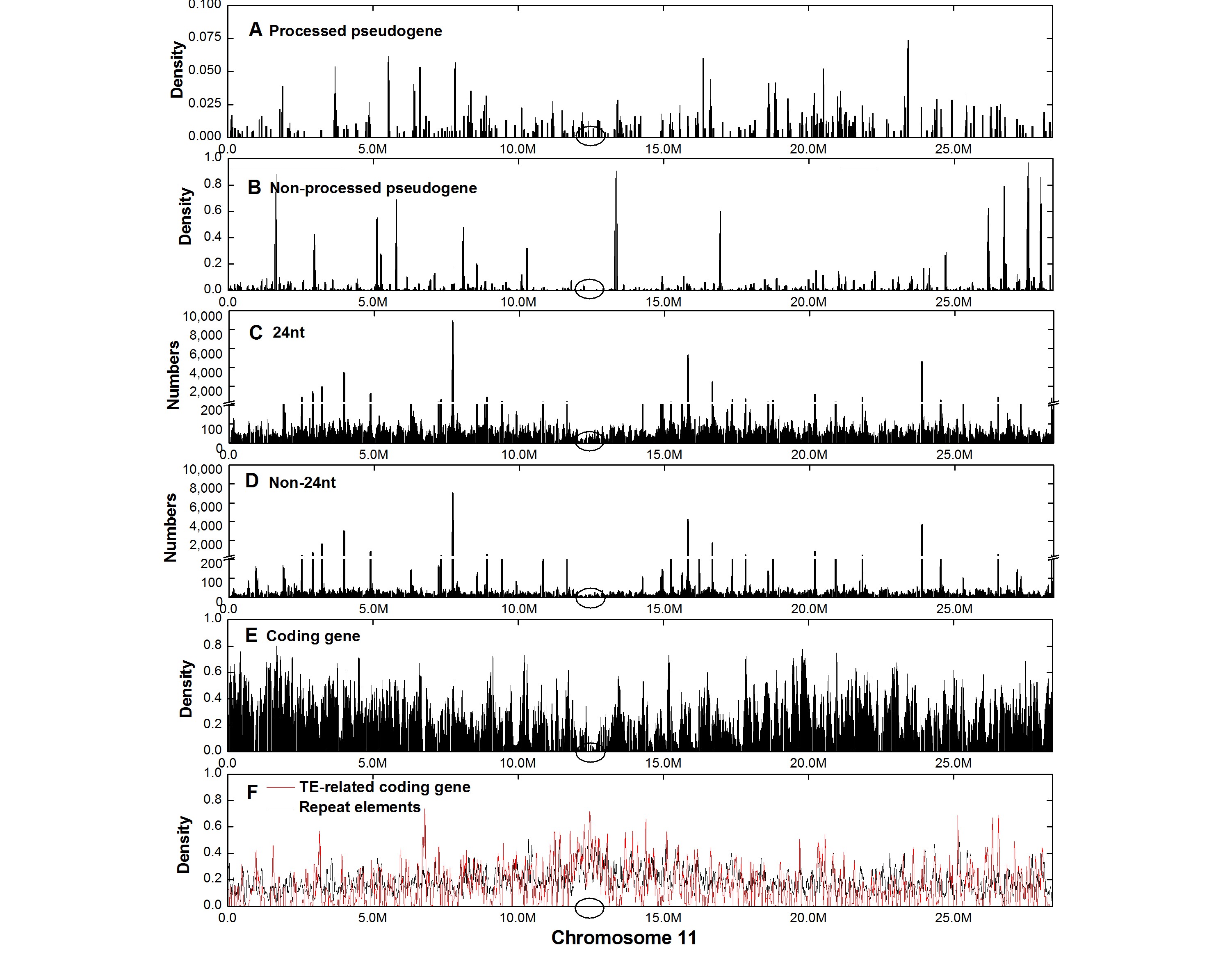


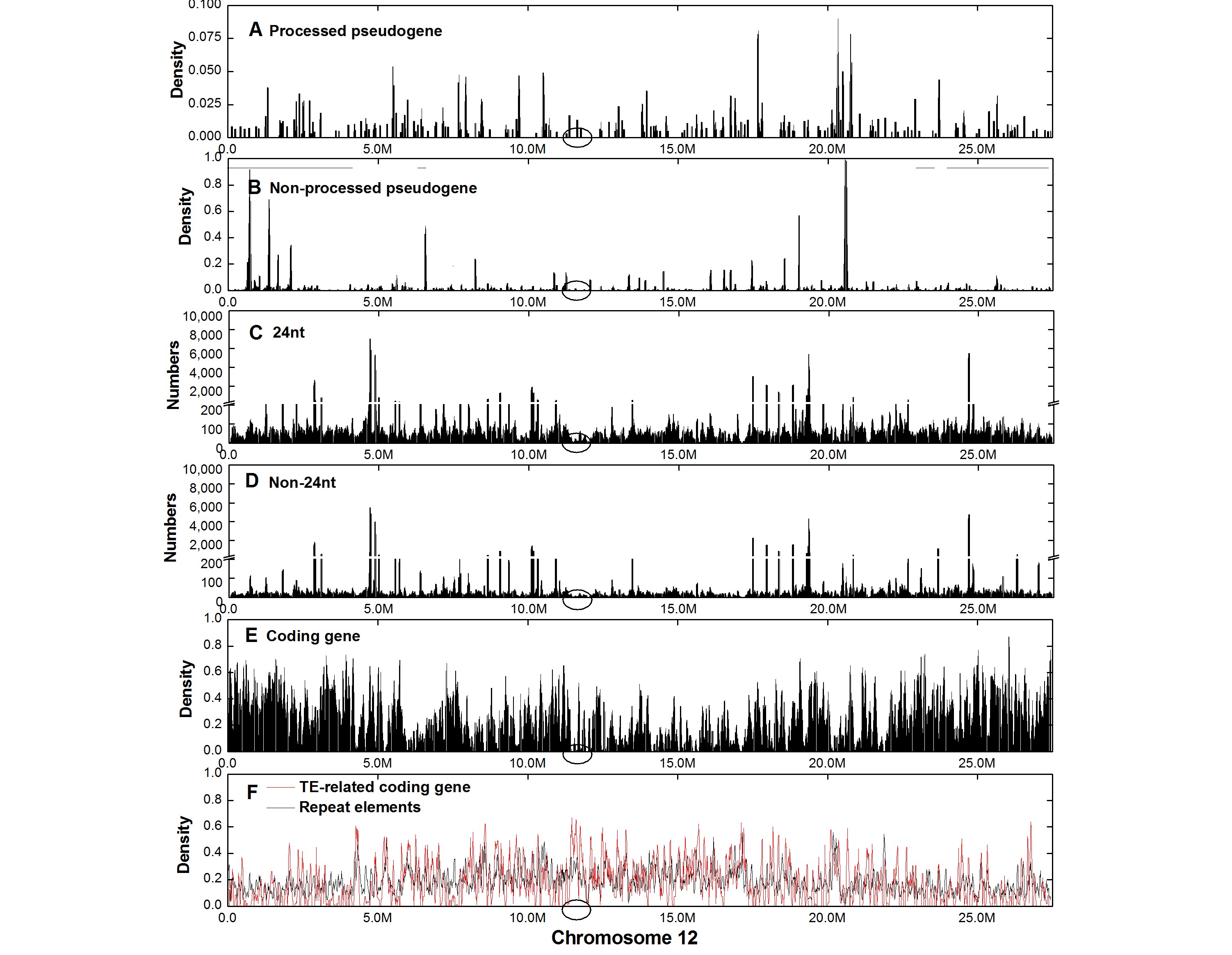

Supplement: Figure S1 — Genome-wide distribution of rice pseudogenes, siRNAs from developing rice grains, and repeats. (10.17 MB DOC) [file pcbi.1000449.s001.doc]
