# Supplementary material for: Small RNAs Originated from Pseudogenes: cis- or trans-Acting?
Source: PLoS Comput Biol. 2009 Jul 31;5(7):e1000449. doi: 10.1371/journal.pcbi.1000449 (PMC2708354; doi:10.1371/journal.pcbi.1000449)
Supplement: Table S1 — Comparison of pseudogenes with antisense siRNAs in different small RNA libraries. (0.07 MB DOC) [file pcbi.1000449.s002.doc]

Small RNAs Originated From Pseudogenes, *cis*- or *trans*-Acting?

Xingyi Guo, Zhaolei Zhang, Mark B. Gerstein, and Deyou Zheng

**Table S1.** Comparison of pseudogenes with antisense siRNAs in different small RNA libraries. This table lists the numbers of pseudogenes with antisense small RNA in developing rice grains that were also detected with at least one, two, three, four or five antisense siRNAs in the five additional libraries (a-e). The four numbers in parentheses refer to overlaps with the results from our primary dataset (i.e., ≤10 DAF developing rice grains) in this sequential order: pseudogenes (2582) of at least one siRNA, pseudogenes (145, group A in Figure 1) with antisense RNA density of 0.04, pseudogenes (38, group B) with siRNAs possibly targeting parents, and pseudogenes (21, a subset of group B) with siRNAs targeting adjacent parents.

| Small RNA Numbers | Numbers of pseudogenes with antisense siRNAs | | | | | |
| --- | --- | --- | --- | --- | --- | --- |
|  | a | b | c | d | e | a+b+c+d+e |
| ≥1 | 1152  (585/91/31/18) | 396  (236/13/3/3) | 1109  (595/48/11/5) | 361  (171/12/4/2) | 33  (15/1/0/0) | 2350  (1134/119/36/19) |
| ≥2 | 281  (199/66/27/17) | 45  (38/2/0/0) | 303  (205/27/6/2) | 46  (23/2/1/1) | 2  (2/1/0/0) | 592  (396/85/31/18) |
| ≥3 | 108  (93/57/24/16) | 12  (11/1/0/0) | 109  (82/20/4/1) | 11  (5/1/1/1) | 1  (1/1/0/0) | 221  (173/73/27/16) |
| ≥4 | 68  (62/49/23/16) | 2  (2/1/0/0) | 51  (40/16/4/1) | 3  (1/0/0/0) | 1  (1/1/0/0) | 119  (100/64/26/16) |
| ≥5 | 51  (51/44/22/15) | 2  (2/1/0/0) | 28  (24/13/3/0) | 3  (1/0/0/0) | 1  (1/1/0/0) | 82  (76/58/25/15) |

1. Dehulled mature grain, 141,370 small RNAs (matching uniquely to rice genome, ditto below). Heisel et al., 2008 (49);
2. 23 days old seedlings, 58,863 small RNAs. Heisel et al., 2008 (49);
3. A mixture of RNAs for MPSS from (1) seedlings treated with ABA, (2) nipponbare immature panicles - 90 days old plants, (3) germinating seedlings infected with Magnaporthe grisea, (4) germinating seedlings, (5) stem, and (6) seedling control for ABA treatment. 136,870 small RNAs. Nobuta et al., 2007 (50);
4. Four-week old seedlings, 73,174 small RNAs. Zhou et al., 2008 ;
5. CRSDB, a mixture of RNAs isolated from 30 to 60 day leaves (~16.5%), 10, 25 and 30 day seedlings (~11%), 4–7 cm inflorescences (~16.5%) and 25 day seedling polysomes (~16.5%), 5,521 small RNAs. Johnson et al., 2006 (51).
